# Multilevel Assessment of Glycemic, Hormonal, and Oxidative Parameters in an Experimental Diabetic Female Rat Model

**DOI:** 10.3390/biomedicines13040922

**Published:** 2025-04-09

**Authors:** Iulian Tătaru, Ioannis Gardikiotis, Oana-Maria Dragostin, Luminita Confederat, Cerasela Gîrd, Alexandra-Simona Zamfir, Ionela Daniela Morariu, Carmen Lidia Chiţescu, Ancuța Dinu (Iacob), Liliana Costea Popescu, Carmen Lăcrămioara Zamfir

**Affiliations:** 1 Department of Morphofunctional Sciences I, “Grigore T. Popa” University of Medicine and Pharmacy, 700115 Iasi, Romania; iulian-alexandru-i-tataru@d.umfiasi.ro (I.T.); carmen.zamfir@umfiasi.ro (C.L.Z.); 2 CEMEX—Advanced Center for Research and Development in Experimental Medicine, “Grigore T. Popa” University of Medicine and Pharmacy, 700454 Iasi, Romania; dr.gardikiotis@yahoo.com; 3 Research Centre in the Medical-Pharmaceutical Field, Department of Pharmaceutical Science, Faculty of Medicine and Pharmacy, “Dunarea de Jos” University of Galati, 800201 Galati, Romania; carmen.chitescu@ugal.ro (C.L.C.); ancuta.dinu@ugal.ro (A.D.); 4 Department of Biomedical Sciences, “Grigore T. Popa” University of Medicine and Pharmacy, 700115 Iași, Romania; luminita.confederat@yahoo.com; 5 Department of Pharmacognosy, Phytochemistry and Phytotherapy, Faculty of Pharmacy, “Carol Davila” University of Medicine and Pharmacy, 050474 Bucharest, Romania; cerasela.gird@umfcd.ro (C.G.); liliana.costea@drd.umfcd.ro (L.C.P.); 6 Department of Medical Sciences I, “Grigore T. Popa” University of Medicine and Pharmacy, 700115 Iasi, Romania; simona-zamfir@umfiasi.ro; 7 Department of Environmental and Food Chemistry, Faculty of Pharmacy, “Grigore T. Popa” University of Medicine and Pharmacy, 700115 Iasi, Romania; ionela.morariu@umfiasi.ro

**Keywords:** diabetes, oxidative stress, hormonal imbalance, alloxan, *Salvia officinalis*, TGF-β1, GPX3, metformin

## Abstract

**Background**: Diabetes mellitus induces profound metabolic and endocrine alterations, impacting reproductive function through oxidative stress and hormonal imbalances. This study investigated the effects of alloxan-induced diabetes on hormonal status and oxidative stress in female Wistar rats. **Methods**: A synthetic sulfonamide derivative (compound S) was obtained via chemical synthesis and characterized by elemental and spectral analysis. *Salvia officinalis* extract was phytochemically profiled using UHPLC-HRMS and assessed for antioxidant potential using DPPH, ABTS, and FRAP assays. The synthetic compound and the plant extract, along with metformin were evaluated in vivo for their potential antihyperglycemic, hormone-regulating, and antioxidant properties., Serum levels of progesterone, estradiol, and follicle-stimulating hormone (FSH) were evaluated alongside oxidative stress biomarkers transforming growth factor-beta 1 (TGF-β1) and glutathione peroxidase 3 (GPX3). **Results**: Diabetic rats (untreated) exhibited a significant decrease in estradiol (22.00 ± 4.1 pg/mL vs. 54.74 ± 17.5 pg/mL in controls, *p* < 0.001) and an increase in progesterone levels (17.38 ± 9.6 ng/mL vs. 3.59 ± 0.90 ng/mL in controls, *p* < 0.05), suggestive for ovarian dysfunction. TGF-β1 levels were elevated in diabetic rats (27.73 ± 19.4 ng/mL vs. 21.55 ± 13.15 ng/mL in controls, *p* < 0.05), while increased serum GPX3 (61.50 ± 11.3 ng/mL vs. 38.20 ± 12.84 ng/mL in controls, *p* < 0.05) indicates enhanced oxidative stress. Statistical analysis revealed a correlation between serum GPX3 levels, FSH (*p* = −0.039), and estradiol (*p* = −0.025) in the diabetic group (L2). **Conclusions**: These findings contribute new evidence regarding the effects of diabetes on reproductive hormones and oxidative stress in female models.

## 1. Introduction

Diabetes mellitus (DM) is a major global health issue, with a dramatic increase in prevalence over the past decades. In 2021, approximately 537 million people (10.5% of the global population) were affected by diabetes, and projections indicate that this number could rise to 643 million by 2030 and 783 million by 2045. Type 2 diabetes (T2D) accounts for 90% of all cases [1,2,3].

Hyperglycemia-induced oxidative stress and inflammation are central mechanisms in metabolic dysfunction and diabetes complications [4,5,6,7]. These pathological changes affect multiple organ systems, including the nervous, circulatory, renal, and reproductive systems, as well as the thyroid and adrenal glands [1,8,9]. The endocrine and metabolic disturbances observed in diabetes involve complex interactions between oxidative stress, chronic inflammation, and hormonal regulation [4,10,11].

Among the key molecular mediators in diabetes pathology, transforming growth factor-beta 1 (TGF-β1) plays a critical role in tissue remodeling, fibrosis, and chronic inflammation [4,12]. TGF-β1 interacts with reactive oxygen species (ROS), further amplifying oxidative stress and pro-inflammatory cytokine activation (IL-6, TNF-α), creating a self-sustaining pathogenic cycle [13]. Elevated TGF-β1 levels were correlated with oxidative stress, extracellular matrix accumulation, and endothelial dysfunction, contributing to vascular complications in diabetes [14,15]. In individuals with type 2 diabetes, altered serum TGF-β1 levels have been associated with higher HbA1c (glycated hemoglobin) and fasting glucose concentrations [16,17].

In the context of the increasing prevalence of metabolic and endocrine disorders, the relationship between glucose metabolism and endocrine function has become a major topic of interest [18,19]. The link between diabetes and reproductive health is increasingly recognized. According to recent studies up to 40% of women with diabetes experience reproductive disorders including menstrual irregularities, pubertal delay, subfertility, polycystic ovary syndrome, hyperandrogenism, and premature menopause [20]. Chronic hyperglycemia disrupts the hypothalamic-pituitary-ovarian axis, leading to menstrual irregularities, ovarian dysfunction, and infertility [9,21,22,23]. Insulin resistance has been recognized as a contributor to the pathogenesis of polycystic ovary syndrome, interacting synergistically with luteinizing hormone (LH) to stimulate ovarian androgen synthesis [21]. Furthermore, insulin has been identified as a factor that downregulates the hepatic synthesis of sex hormone-binding globulin (SHBG) in the liver, leading to elevated levels of free testosterone which contributes to ovulatory dysfunction [24,25].

Animal models are widely employed as essential tools for understanding diabetes pathophysiology including induced ovarian dysfunction [26]. However, most in vivo studies on diabetes-induced reproductive dysfunction focus on male fertility, with limited data on the effects of diabetes on ovarian function and endocrine regulation in females [27,28,29]. To address this gap, our in vivo study on alloxan-induced diabetes in rats aims to provide insights into the pathophysiological mechanisms affecting female reproductive function.

Given the intricate crosslink between glucose metabolism, oxidative stress, and hormonal homeostasis, it is important to evaluate potential therapeutic agents with both antihyperglycemic and antioxidative properties. In this context, the present study evaluated three therapeutic interventions: metformin, a widely used first-line antidiabetic agent with documented reproductive function-regulating effects [30], used as a positive control; a compound derived from a sulfonamide—*p*-toluenesulfonamide (compound S), structurally related to known antidiabetic sulfonamides; and *Salvia officinalis* extract, a plant with traditional use in metabolic disorders and reported antioxidant properties in experimental animals models [31].

Considering previous studies that have reported the benefits of using certain natural compounds, either alone or in combination with synthetic agents [32,33], *Salvia officinalis* extract was included as a treatment in two experimental groups of our in vivo study.

In this context, the present study aims to investigate the effects of diabetes metabolic and oxidative stress on female reproductive function using an in vivo model of alloxan-induced diabetes in Wistar rats. Female subjects were considered to provide a more appropriate model for assessing the reproductive endocrine disruption induced by hyperglycemia and oxidative stress. This decision aligns with the increasing emphasis on sex-specific analysis in biomedical research, particularly involving endocrine and metabolic pathways [34]. The study evaluated the impact of diabetes on ovarian function, analyzing serum levels of progesterone, estradiol, and follicle-stimulating hormone (FSH) as key reproductive markers as well as the role of oxidative stress in endocrine dysregulation, assessing TGF-β1 and glutathione peroxidase 3 (GPX3) as biomarkers of oxidative imbalance [35]. The therapeutic potential of a novel synthetic compound and *Salvia officinalis* extract, in restoring hormonal homeostasis and reducing oxidative stress, was considered.

## 2. Materials and Methods

### 2.1. Antidiabetic Compound Synthesis and Confirmation

A chemical synthesis method was employed, using the following chemical reagents: *p*-toluenesulfonamide 98% (Sigma Aldrich, St. Louis, MO, USA), urea 99.98% (Merck, Readington, NJ, USA), potassium carbonate K_2_CO_3_, 98% (Supleco, Bellefonte, PA, USA), and acetone 99.9% (Supleco). The synthesis was conducted using a reflux system (BIOBASE Biodustry Shandong Co., Ltd., Jinan, China).

The synthesis of *N*-(diaminomethylene)-4-methylbenzenesulfonamide (compound S) was carried out by reacting *p*-toluenesulfonamide with urea in the presence of potassium carbonate (K_2_CO_3_) at 75 °C for 6 h. The water generated during the reaction was removed.

For structural confirmation of the synthesized compound, a Vanquish Flex UHPLC system coupled with a high-resolution Orbitrap Exploris 120 mass spectrometer (Thermo Fisher Scientific, Waltham, MA, USA) was used in full MS mode, operating at a resolution of 120,000 (FWHM) at *m*/*z* 200. The mobile phase consisted of ultrapure water with 0.1% formic acid (98–100%, LC-MS grade) and methanol with 0.1% formic acid (98–100%, LC-MS grade). Compound detection was performed by targeting the exact mass within the total ion current (TIC) scan, using a mass window of 2 ppm.

### 2.2. Extraction and Characterization of the Salvia officinalis Extract

The plant material was purchased as single-component medicinal teas from pharmaceutical units in Romania. The dried plant material was ground to achieve a uniform particle size. Extraction was performed using a liquid-solid method, employing 50% ethanol (Merck) as the solvent, with a plant material-to-solvent ratio of 1:10 (g/mL). The extraction process was conducted under reflux for 30 min and involved a two-step successive extraction, after which the filtrates were combined and concentrated under vacuum at temperatures ≤ 40 °C to remove excess solvent. The final concentration was achieved through lyophilization to obtain a dry extract while preserving the integrity of the bioactive compounds. The resulting extract appeared as a dry, uniformly dispersed powder, maintaining the organoleptic properties characteristic of the plant source.

The content of flavonoids (via chelation with AlCl_3_), phenolic acids (via nitroso derivatives formed in the Arnow reagent reaction), and total polyphenols (via molybdenum Mo^4+^ and Mo^+^ derivatives formed in the Folin-Ciocâlteu reaction) was determined spectrophotometrically (Jasco V-530 spectrophotometer, Tokyo, Japan) following recent literature methodologies [36]. Experiments were conducted in four independent replicates, and results were expressed as mean values (g/100 g dry extract) ± standard deviation. The quantification was performed using chlorogenic acid as a reference standard for total phenolic acids, rutin for total flavonoids, and tannic acid for total polyphenols. Calibration curves of absorbance versus concentration were plotted for these standards within the following concentration ranges: 10–60 mg/mL for chlorogenic acid and rutin, and 1–10 µg/mL for tannic acid.

The antioxidant activity of the extract was assessed using three spectrophotometric methods: FRAP (Ferric Reducing Antioxidant Power), evaluating iron ion reduction capacity; DPPH (2,2-diphenyl-1-picrylhydrazyl)—measuring the ability to neutralize free radicals; ABTS (2,2′-azinobis(3-ethylbenzothiazoline-6-sulfonic acid))—determining the neutralization of ABTS^+^ radicals, following literature protocols [36].

Based on the obtained values, inhibition curves (%) as a function of concentration (mg/mL) were constructed for ten different concentrations of the tested extract. Using the corresponding linear equations, IC_50_ (Inhibitory Concentration 50, mg/mL) values were determined for the ABTS and DPPH methods, while EC_50_ (Effective Concentration 50) was determined for the FRAP method (for y = 50), for both the reference compound (ascorbic acid) and the tested extracts. Details of the analytical methods including the calibration curves (Figures S1–S3) are available in Supplementary Materials.

To identify and quantify potential bioactive polyphenolic compounds in the sage extract, ultra-high-performance liquid chromatography coupled with high-resolution mass spectrometry (UHPLC-HRMS/MS) was employed, using the Q-Orbitrap-MS technique (Thermo Fisher Scientific). Chromatographic separation was achieved using an Acquity U-HPLC C18 column (Waters, Milford, MA, USA) with a mobile phase consisting of water: metanol, containing 100 µL/L formic acid (LC-MS grade), in a gradient mode over 26 min. A heated electrospray ionization (HESI) source was used in negative ion mode for ionization.

Sample preparation involved dissolving the lyophilized extract in 1 mL dimethyl sulfoxide (DMSO) and diluted to a concentration of 1 mg/mL with methanol. A dilution to 1:10 (*v*/*v*) with 10% methanol was performed before injection in the UHPLC-HRMS system.

Data acquisition was carried out in variable data-independent acquisition (vDIA) mode, including full-scan MS at a resolution of 70,000 FWHM at *m*/*z* 200, along with simultaneous MS/MS analysis at a resolution of 35,000 (*m*/*z* 200). The MS/MS analysis was divided into five successive MS^2^ scanning events, covering the 100–1000 *m*/*z* mass range.

For compounds with no analytical standards available, an in-house spectral library was used for the identification of the compounds in the total ion current (TIC) spectra. Additionally, for unknown compounds, the most plausible molecular formula with minimal mass error was searched in the ChemSpider database. Given the structural similarities between flavones, isoflavones, and phenolic acids, the MS/MS ion fragments were used for structural confirmation through spectral databases, including mzCloud™, AdvancedMass, and PubChem, according to the literature [37].

### 2.3. In-Vitro Testing. Study Design

#### 2.3.1. Used Substances

For metformin hydrochloride and alloxan, the analytical standard was supplied by Alfa Aesar (Thermo Fisher Scientific, Haverhill, MA, USA). Alloxan was formulated as a solution in physiological saline (20 mg/mL). The active substances, metformin and the synthetically obtained compound (S), described in the previous chapter, were formulated as suspensions in 1% carboxymethyl cellulose (CMC) at concentrations of 25 mg/mL for metformin and 75 mg/mL for compound S. The sage extract was also formulated as a suspension (75 mg/mL) in 1% CMC. The suspensions were distributed in amber glass bottles and stored at 4 °C throughout the administration period.

#### 2.3.2. Animals

A total of 42 Wistar rats were used in the experiment, obtained through the “Gr. T. Popa” University of Medicine and Pharmacy, Iași, from the Cantacuzino Institute, Bucharest. The animals were housed at the CEMEX biobase within the “Gr. T. Popa” University of Medicine and Pharmacy, Iași, throughout the study period.

All experimental procedures adhered to international ethical regulations and were approved by the Medical Ethics Committee of “Gr. T. Popa” University of Medicine and Pharmacy, Iași, Romania (Approval No. 169/22.03.2022). Additionally, the experiment complied with the Guidelines on the Care and Use of Animals for Scientific Purposes (National Advisory Committee for Laboratory Animal Research, 2004). The research methodology conformed to national and international standards (Law No. 206/27 May 2004, EU Directive 2010/63/EU on animal experimentation, and CE86/609/EEC).

Throughout the experiment, standard laboratory conditions were maintained, including a temperature of 20–22 °C, light cycle (12 h of natural light per day), and air renewal every 24 h. Cages hygiene was maintained by cleaning every 24 h, including the replacement of bedding and food/water containers. A balanced diet and ad libitum access to food and water were provided. No direct contact with other species ensured the isolation of the animals.

Before the start of the experiment, the rats underwent a 7-day acclimatization period, during which they were kept under standardized laboratory conditions. This step was essential to reduce transport-induced stress and facilitate adaptation to the experimental environment, in accordance with international guidelines on laboratory animal welfare. No quantitative biomarkers of stress were measured during the acclimatization phase. However, all animals were monitored daily for signs of stress or discomfort based on standard qualitative criteria, including changes in posture, grooming behavior, locomotor activity, food and water intake, and fur condition.

#### 2.3.3. Experimental Groups and Diabetes Induction

The rats were divided into six experimental groups, each consisting of seven animals, assigned based on the presence or absence of diabetes and the administered treatment:

- Group 1 (L1)—Untreated control
- Group 2 (L2)—Diabetes-induced, untreated
- Group 3 (L3)—Diabetes-induced, treated with metformin (300 mg/kg b.w./day)
- Group 4 (L4)—Diabetes-induced, treated with the synthetic compound (S) (150 mg/kg b.w./day)
- Group 5 (L5)—Diabetes-induced, treated with sage extract (150 mg/kg b.w./day)
- Group 6 (L6)—Diabetes-induced, treated with both the synthetic compound and sage extract (150 mg/kg b.w./day each)

The selected dose of metformin (300 mg/kg body weight) falls within the range commonly used in rodent studies assessing antidiabetic effect [38,39]. For compound S, a dose of 150 mg/kg was established, representing less than 1/10 of the reported median lethal dose (LD_50_ = 2000 mg/kg), consistent with safety margins applied in subacute toxicity protocols [40].

The dose of alloxan was established based on data reported in the literature, which indicates that in rats, effective diabetogenic doses typically range from 40 to 200 mg/kg [41]. A dose of 150 mg/kg body weight was selected in this study to provide a reliable induction of sustained hyperglycemia, as confirmed in previous rodent models [42].

Before alloxan administration, the rats were 10–12 weeks old, with body weights ranging from 177 to 282 g (mean weight: 213.3 ± 28.2 g) and baseline blood glucose levels between 94 and 114 mg/dL (mean: 100.09 ± 10.8 mg/dL).

Diabetes was induced in groups 2–6 by intraperitoneal injection of alloxan in a single dose of 150 mg/kg body weight in the second week of the study. Since alloxan induces a four-phase glycemic response, with blood glucose levels stabilizing after approximately 200 h [43], glycemic control was performed five days after administration by collecting venous blood from the tail.

A blood glucose threshold of 200 mg/dL was set as the criterion for confirming severe hyperglycemia and significant metabolic alterations indicative of experimental diabetes. As approximately 30% of the rats had glucose levels below this threshold, a second alloxan administration (at the same single dose) was performed at the beginning of the third week. Blood glucose measurement five days later confirmed diabetes induction in all animals in groups 2–6.

The treatments, including metformin, synthetic compound (S), and sage extract, were administered orally via gavage, once daily, at the specified doses, starting from week 4 of the experiment and continuing for five weeks.

#### 2.3.4. Monitoring During the Study

Throughout the eight-week experiment, the animals were monitored weekly to assess both their general condition and key physiological parameters. Clinical evaluations were performed to identify signs of discomfort, distress, or behavioral changes. The clinical examination included observations of appetite, physical activity, fur appearance, and signs of dehydration or inflammation.

Each animal was weighed weekly using a high-precision electronic scale, with recorded values providing insights into the nutritional status and the metabolic impact of induced diabetes. Simultaneously, blood glucose levels were measured by collecting a small peripheral blood sample from the tail and analyzing it with a portable glucometer. These measurements allowed for the monitoring of the diabetogenic model’s effectiveness and the glycemic variations induced by the administered treatments.

At the end of the study, animals were anesthetized with ketamine (75 mg/kg, intraperitoneally) for blood sample collection. Due to the non-recovery nature of the procedure, no analgesia was required post-anesthesia. Euthanasia was performed via overdose of thiopental sodium (≥150 mg/kg, i.p.), in accordance with the institutional guidelines and Directive 2010/63/EU for the protection of animals used for scientific purposes [44].

#### 2.3.5. Collection of Biological Samples and Conducted Analyses

Blood samples were collected in anticoagulant-coated tubes (e.g., EDTA) and stored at −20 °C until analysis. To minimize variability, all sample collections were performed by the same investigator, using identical instruments and at consistent time intervals.

#### 2.3.6. Biochemical and Hormonal Analyses

Serum levels of progesterone, estradiol, luteinizing hormone (LH), and follicle-stimulating hormone (FSH) were analyzed using chemiluminescence immunoassay techniques, employing specific kits (ADVIA Centaur, Siemens Inc., Malvern, PA, USA) following the manufacturer’s protocol. The quantification limits of the test are as follows: FSH, 0.3 mIU/mL; estradiol, 11.8 pg/mL; LH, 0.07 mIU/mL; Progesterone, 0.23 ng/mL.

TGF-β1 and glutathione peroxidase (GPX3) markers were determined using ELISA immunoassay and spectrophotometric techniques with specific kits (BioVendor, Brno, Czech Republic). The detection limit of TGF-β1 8.6 pg/mL ensured precise measurement of low concentrations. The detection limit of GPX3 was 100 pg/mL. The method detects only GPX3, with no cross-reactivity with other GPX isoforms (e.g., GPX1, GPX2, GPX4).

Details related to the analytical methods are presented in the Supplementary Materials.

### 2.4. Statistical Methods

Statistical analysis was performed using XLSTAT 2023.3.0 (Basic+) software. The normality of data distribution was assessed using the Shapiro-Wilk test. Levene’s test was used to assess variance homogeneity among groups. To compare datasets with different magnitude orders, Z-score normalization was performed, enabling a comparable analysis across variables.

Correlation analyses were performed using Pearson’s correlation coefficient (for normally distributed data) and Spearman’s rank correlation (for non-normally distributed data). Statistical significance was determined through one-way analysis of variance (ANOVA) for parametric data, followed by Duncan’s multiple range test for post-hoc comparisons and non-parametric Kruskal-Wallis tests followed by Dunn’s test for multiple comparisons with the control group.

To explore data patterns and relationships among experimental parameters, correlation tests, principal component analysis (PCA), and hierarchical cluster analysis (HCA) were conducted. Additionally, linear and multiple regression analyses were applied to investigate potential predictive relationships between variables.

Data are presented as mean ± standard deviation (SD) unless otherwise specified.

## 3. Results

### 3.1. Structural Confirmation of the Sulfonamide Derivative and Antioxidant Activity In Vitro Test

**Figure 1:**
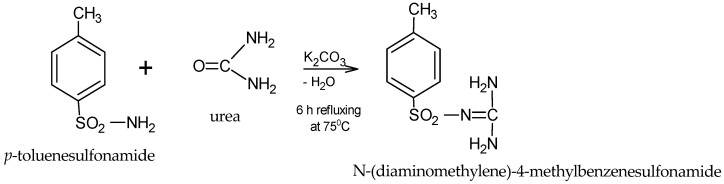
General scheme for obtaining the sulfonamide.

The structure of the synthesized product (C_8_H_11_O_2_N_3_S, exact mass 213.0572), is presented in Figure 1.

**Figure 2:**
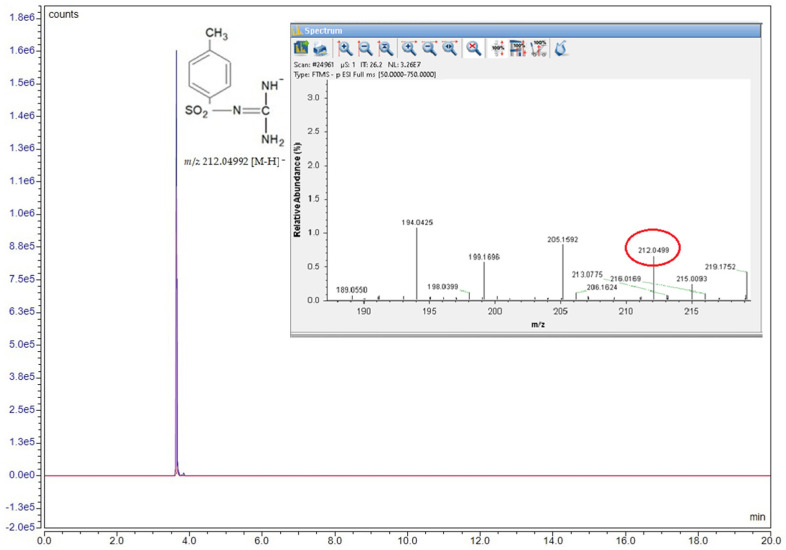
Structural formula, chromatogram, and MS spectrum of the compound *N*-(diaminomethylene)-4-methylbenzenesulfonamide.

The sulfonamide derivative synthesis was structurally confirmed by the HRMS method, and the corresponding spectrum is presented in Figure 2. The compound was diluted in methanol (100 ng/mL) and then directly analyzed by the mass detector. Detection was carried out in negative ionization mode, with the selected ion having a mass of *m*/*z* 212.04992 [M-H]⁻.

The antioxidant activity of the synthetic sulfornamide (S) was assessed using two spectrophotometric methods: FRAP and DPPH. The results show moderate antioxidant activity according to the DPPH method (IC_50_ = 0.018 mg/mL) and no activity according to the FRAP method. Details related to analytic methodology and calibration curves (Figure S4) are available in Supplementary Materials.

### 3.2. In Vitro Testing of Antioxidant Effect of the Salvia officinalis Extract

The antioxidant activity profiles of the tested extract are correlated to the chemical nature and concentration of individual secondary metabolites (Table 1). The assay procedures differ in sensitivity to specific antioxidant classes, reaction kinetics, and pH conditions, due to different reaction mechanisms, which may lead to method-specific variations in antioxidant capacity.

The data presented in Table 2 shows the correlation between the used methodologies (DPPH, ABTS, and FRAP) for the evaluation of the antioxidant activity in the sage extracts. The Pearson coefficient (r) calculated for each set of data pairs displayed values above 0.900, which presents a very strong correlation between the experimental methodologies.

Although based on different redox mechanisms, and different methodologies, the antioxidant assays used (DPPH, ABTS, FRAP) showed strongly correlated IC_50_/EC_50_ values (Pearson r > 0.90), confirming the complementarity and mutual validation of the results. Total flavonoids and total polyphenols do not show statistically significant correlations with IC_50_ or EC_50_, suggesting that these compounds are not the main predictors of antioxidant activity in this dataset. This may be due to the non-specific nature of the Folin–Ciocâlteu assay, which also detects other reducing substances. On the other hand, the antioxidant capacity depends not only on total quantity, but also on the chemical structure, redox potential, and interaction of specific polyphenolic compounds. However, phenolic acids are strongly positively correlated with IC_50_ (ABTS, DPPH), which could indicate that these compounds play a specific role in reducing the antioxidant activity measured by these methods.

### 3.3. Identification of Potentially Active Compound in Salvia officinalis Extract

A UHPLC-HRMS-MS analysis of *Salvia officinalis* extract identified a total of 54 polyphenolic compounds, of which 24 were quantified. Table 3 summarizes the monitored ion, the retention time, and the amount of the potentially bioactive compounds in sage extract.

For the compounds with no available analytical standards, the presumptive confirmation of the identity was done by high-resolution MS-MS analysis and comparison of fragmentation patterns with spectra databases. The monitored ion and the fragments used for confirmation are presented in Table S1.

The detected compounds belong to three main chemical classes: flavonoids and their glycosides (luteolin, kaempferol, quercetin, rutin, apigenin), phenolic and phenolcarboxylic acids (rosmarinic acid, chlorogenic acid, ferulic acid, caffeic acid, ellagic acid, and gallic acid). Due to high-resolution technical capabilities, in addition to common polyphenols, specific compounds of *Salvia officinalis* have been identified, including terpenoids such as carnosic acid, carnosol, and ursolic acid, as well as phenolic acids like salvianolic acids (A, B, C), rosmarinic acid, and its derivatives (Figures S5 and S6).

**Figure 3:**
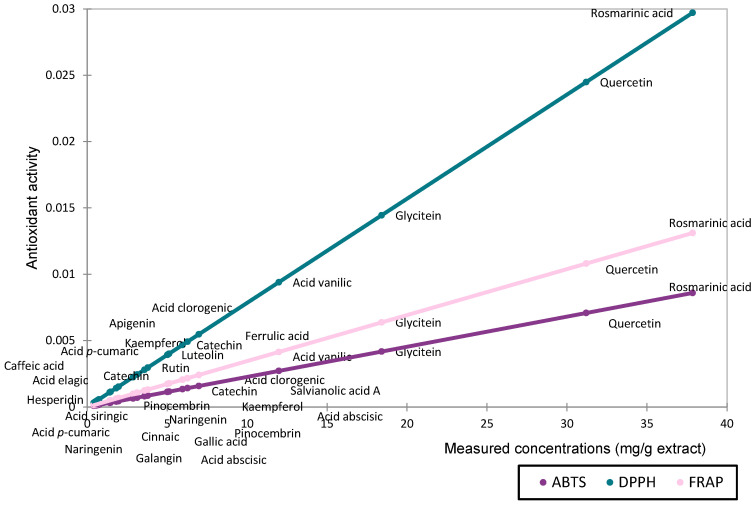
Scatter plot representing the contribution of the determined compounds to antioxidant activity based on concentration (mg/g extract).

The relationship between the compound quantities (mg/g extract) and antioxidant activity (IC_50_/EC_50_) was analyzed using a scatter plot (Figure 3).

The antioxidant activity assessed by DPPH, ABTS, and FRAP followed comparable trends for compounds present in high concentrations. However, methodological differences became more apparent when evaluating compounds at lower concentrations. The DPPH method appears to be more sensitive in detecting the antioxidant activity of major compounds. Based on the measured quantities, rosmarinic acid, quercetin, and glycitein are the main contributors to the antioxidant activity of the extract.

### 3.4. In Vivo Testing

#### 3.4.1. Effect on Body Weight

**Figure 4:**
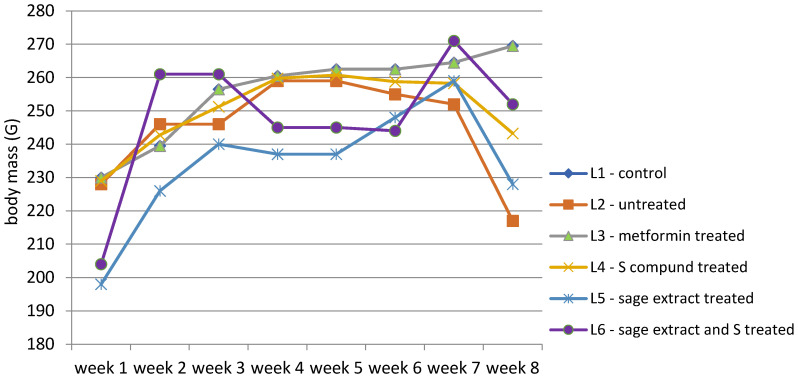
Evolution of body weight (g) expressed as the group median throughout the study.

The body weight of all groups was measured according to the experimental protocol. The evolution of body weight across the eight-week experimental period for all six groups is illustrated in Figure 4, with detailed weekly values provided in Table S2 (Supplementary Materials).

The evolution of body weight across the eight-week experimental period highlights distinct patterns among the experimental groups (Table S2). The control group (L1) exhibited a consistent and gradual increase in body weight, from a median of 230 g in week 1 to 269.5 g in week 8. In contrast, the diabetes-induced untreated group (L2) followed a similar pattern up to week 3 but exhibited marked weight loss following diabetes onset, reaching a minimum of 217 g by week 8. The metformin-treated group (L3) followed a similar trend with L1, with stable median weights from week 4 onward (260.5 g to 269.5 g). The group receiving the synthetic compound (L4) showed initial stabilization, but following treatment onset, body weight gradually decreased (from 259 g to 243.25 g), with no recovery trend. Subjects treated with sage extract alone (L5) experienced moderate improvement, showing more stable weight than untreated animals, but did not reach the levels observed in either the control or metformin groups. Finally, the group receiving combined treatment (L6: sage extract + compound) showed a substantial increase in body weight after the treatment unset with a slight weight decrease (from 261 g to 252 g), in week 8.

**Figure 5:**
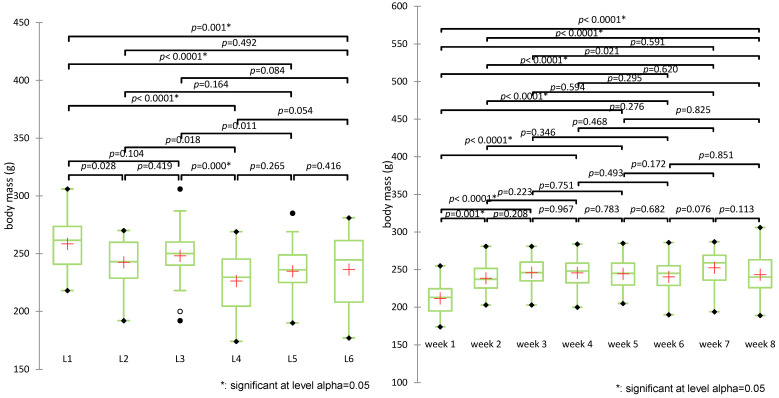
Comparative analysis of body weight evolution over time and across experimental groups (+: mean value of each group).

Data distribution was assessed using multiple normality tests, including Shapiro-Wilk. Results indicated that body weight data were normally distributed for groups L1 to L5 (*p*-values from 0.240 to 0.579), whereas group L6 significantly deviated from normality (*p* = 0.016). While Levene’s test indicated homogeneous variances for most weeks, heteroscedasticity was observed during weeks 2 and 3 (*p* < 0.05). According to these findings, further statistical comparisons of body weight between groups and between weeks of study were performed using non-parametric methods Kruskal-Wallis test followed by Dunn’s post-hoc comparisons (Figure 5).

The Kruskal-Wallis test revealed statistically significant differences in body weight across experimental groups (H(5) = 32.688, *p* < 0.0001). Post-hoc pairwise comparisons using Dunn’s test with Bonferroni correction identified several significant contrasts between groups. The control group (L1) showed significantly higher body weight compared to all other groups (*p* < 0.005). The untreated diabetic group (L2) differed significantly from L4 (*p* = 0.018) but not from other groups. The metformin group (L3) had significantly higher weights than L4 (*p* = 0.000) and was significantly different from L5 (*p* = 0.011). L4 (treated with the synthetic compound alone) showed the lowest mean and rank values, indicating the most pronounced weight reduction.

The groups treated with sage extract alone (L5) or combined (L6) did not show statistically significant differences compared to each other (*p* = 0.416) or to L3 (*p* = 0.086), although both showed a modest increase in weight compared to L4. The combined treatment group (L6) displayed a trend toward weight preservation (mean 236.2 g).

Group clustering by rank averages placed L4 alone in group A, L5, and L6 in group B, and the control (L1), metformin (L3), and untreated (L2) in group C, highlighting the distinct weight response patterns.

Evolution of body weight (g) over the experiment period revealed statistically significant differences between weeks (H(7) = 39.499, *p* < 0.0001), particularly between week 1 and all subsequent weeks (*p* < 0.001), confirming the impact of diabetes induction. Differences were no longer significant after week 3, suggesting stabilization post-intervention.

#### 3.4.2. Effect on Blood Glucose Levels

Blood glucose levels for all subjects were measured weekly using a glucometer, according to the study protocol. The control group (L1) maintained its blood glucose levels within the physiological range (between 95.8–116.2 mg/dL). Starting from the second week, group (L2) and the treated groups (L3–L6) exhibited a significant increase in blood glucose levels, rising from 102 mg/dL (week 1) to 398–516 mg/dL (week 3), indicating that all rats developed hyperglycemia before treatment administration (Table S3).

From week 4 onwards, treatments were administered, and changes in blood glucose levels were observed. In group L3, treated with metformin, blood glucose levels decreased significantly, dropping from 348 mg/dL (week 3) to 224 mg/dL (week 8), although levels remained above normal.

**Figure 6:**
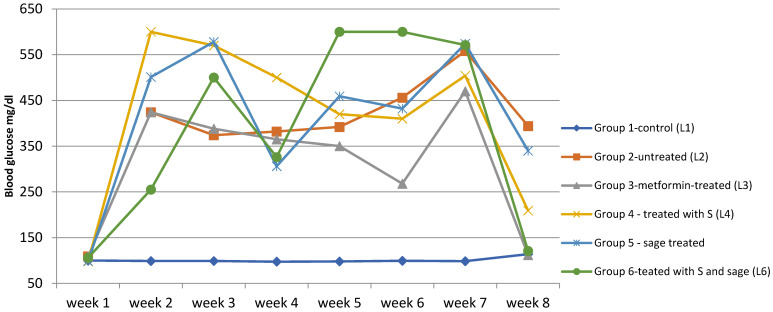
The evolution of blood glucose levels over time in the study groups. Temporal evolution of blood glucose levels (mg/dL) in experimental groups over the 8-week study period. Data are presented as median values.

The synthetic compound (group L4) demonstrated lower efficacy compared to metformin. In group L5, treated with sage extract, the treatment effect was mild and slow, with a gradual decrease in mean blood glucose levels from 423 mg/dL to 358 mg/dL. The treatment applied to group L6 (synthetic compound + sage extract) exhibited the most significant impact on blood glucose levels, leading to a reduction from 550 mg/dL (week 3) to only 139 mg/dL (week 8). Figure 6.

**Figure 7:**
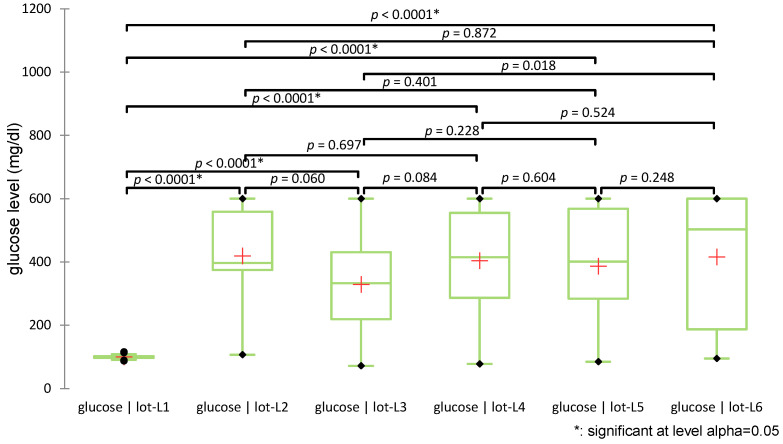
Distribution of glucose values across groups (+: mean value of each group).

The Shapiro-Wilk test, used to assess whether the data distribution is normal, revealed deviations from normality in multiple groups, suggesting high variability in glycemic response (e.g., week 7, group L6, *p* = 0.011; week 4, group L, *p* = 0.013; week 2, group L4, *p* = 0.021). Levene’s test for homoscedasticity showed significant differences in variances between groups for weeks 2 to 8, further justifying the use of the non-parametric Kruskal–Wallis test, followed by pairwise comparisons using Dunn’s procedure with Bonferroni correction for group comparisons (Figure 7).

The test revealed statistically significant differences among groups (H(5) = 66.028, *p* < 0.0001). Post-hoc pairwise comparisons using Dunn’s test with Bonferroni correction indicated that all diabetic groups (L2–L6) had significantly higher glucose levels compared to the non-diabetic control group (L1), confirming diabetes induction (all *p* < 0.0001). No significant differences in glucose levels were observed among the treated groups (L3–L6). Figure 7 illustrates the distribution of glucose values across groups using box plots and indicates the significance levels of pairwise comparisons.

**Figure 8:**
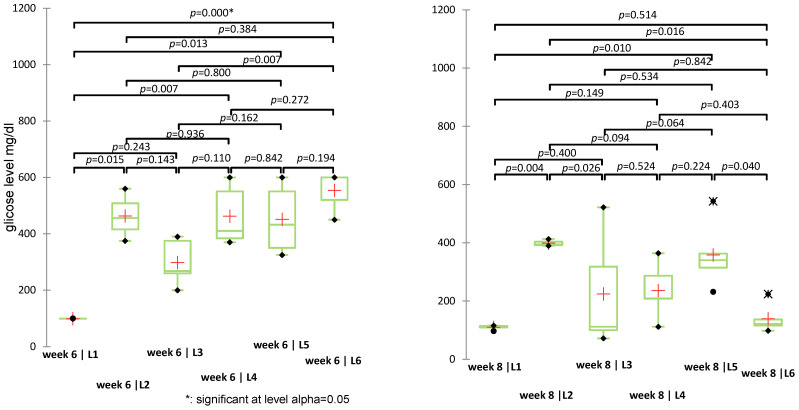
Non-parametric Kruskal-Wallis test for blood glucose values recorded in weeks 6 and 8 (*p*-values < 0.05 indicate statistically significant differences); (+: mean value of each group).

Statistically significant differences were identified in weeks 2 (*p* = 0.032), 3 (*p* = 0.004), 5 (*p* = 0.002), 6 (*p* = 0.004), and 8 (*p* = 0.020), while differences in weeks 1, 4, and 7 were not significant (*p* > 0.05). Pairwise significant differences were observed also between the treatment groups. For example (Figure 8), in week 6, significant differences were observed between L1 (control) vs. L6 (S + sage) with *p* < 0.0001, L3 (metformin) vs. L6 with *p* = 0.007, L1 vs. L5 (sage) with *p* = 0.013. In week 8, L1 vs. L2 (diabetic untreated) showed a significant increase in glycemia (*p* = 0.004), while the S + sage combination (L6) significantly reduced glucose levels compared to L2 (*p* = 0.016) and L5 (sage alone) (*p* = 0.040). Significant intergroup differences (*p* < 0.0033) were identified particularly in weeks 3, 4, and 5, particularly involving the diabetic control group (L1) versus treated groups (L5/L6). A complete list of pairwise comparisons and adjusted *p*-values is provided in Supplementary Table S4.

**Figure 9:**
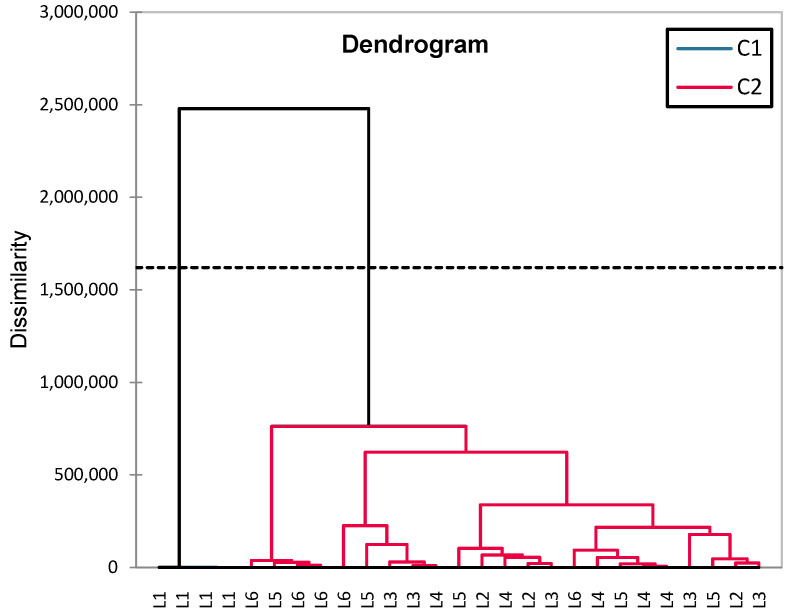
HCA dendrogram (Ward) for analyzing blood glucose similarities between experimental groups (L1–L6).

In addition to classical inferential statistics, hierarchical clustering analysis (HCA) was applied to explore similarities in blood glucose level evolution across groups (L1–L6). This multivariate approach enabled the identification of treatment-related clustering patterns, offering complementary insight into group-level response profiles (Figure 9).

The groups are clustered into two major categories (C1 and C2). The greatest dissimilarity is observed between L1 (control) and the rest of the groups, confirming significant differences between healthy rats and those with induced diabetes (treated or untreated). The diabetes-induced groups (L2–L6) are clustered together, but within this cluster, there are subgroups: L6 (synthetic compound + sage) and L5 (sage) are closely related, suggesting a common influence of sage. L3 (metformin) and L4 (a synthetic compound) are distinct but not far apart, indicating similar effects. L2 (untreated diabetic group) is positioned further from the treated groups, confirming that treatment had significant effects on blood glucose levels.

Statistical analysis using the Aligned Rank Transform (ART) ANOVA was used to investigate the independent and combined effects of the synthetic compound (S) and sage extract on glucose levels during the treatment period (weeks 4 to 8). The results revealed statistically significant main effects for both S (F = 10.10, *p* = 0.0022) and sage (F = 15.80, *p* = 0.0002), indicating that each treatment contributed independently to reducing glucose concentrations. However, the interaction term between S and sage (F = 0.63, *p* = 0.431) was not statistically significant, suggesting no synergistic effect.

#### 3.4.3. Effect on Biochemical Parameters

To evaluate the effects of diabetes on the reproductive system of the study subjects, hormonal assessments were conducted, including measurements of progesterone, FSH, and estradiol, as well as an oxidative stress status evaluation through the analysis of TGF-β1 and GPX3. The analyses were performed at the end of the experiment, using blood samples collected before euthanasia, following the study protocol. The mean values and standard deviations for each group are summarized in Table 4.

For 17 subjects, representing 56.5% of the total subjects at the end of the experiment (n = 30), FSH levels were undetectable. It is worth noting that in group 2 (untreated), FSH values were detectable in all subjects, although the mean value of this parameter was lower than that observed in group 3 (treated with metformin).

**Figure 10:**
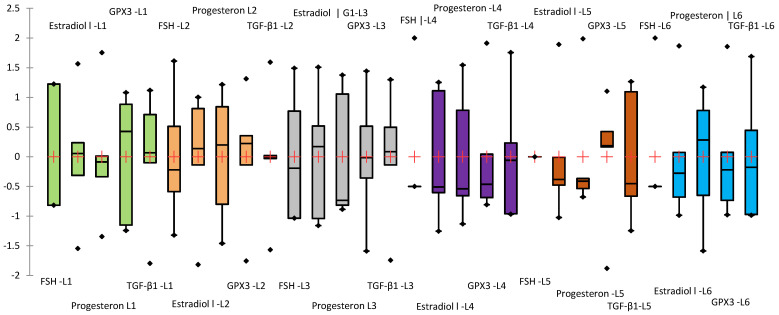
Box-plot representing the standardized mean values (Z-score) of the analyzed biochemical parameters (parameters order in the figure: FSH mU/mL; Estradiol pg/mL; Progesterone ng/mL; GPX3 (ng/mL); TGF-β1 (ng/mL)). The groups were highlighted in different colors: green (L1), orange (L2), gray (L3), violet (L4), brown(L5), and blue (L6).

Since the biochemical parameters analyzed have different magnitudes, a Z-score standardization was applied for graphical representation, allowing for comparative visualization across groups. In this approach, values are expressed relative to the standard deviation from the mean of each variable (Figure 10).

**Figure 11:**
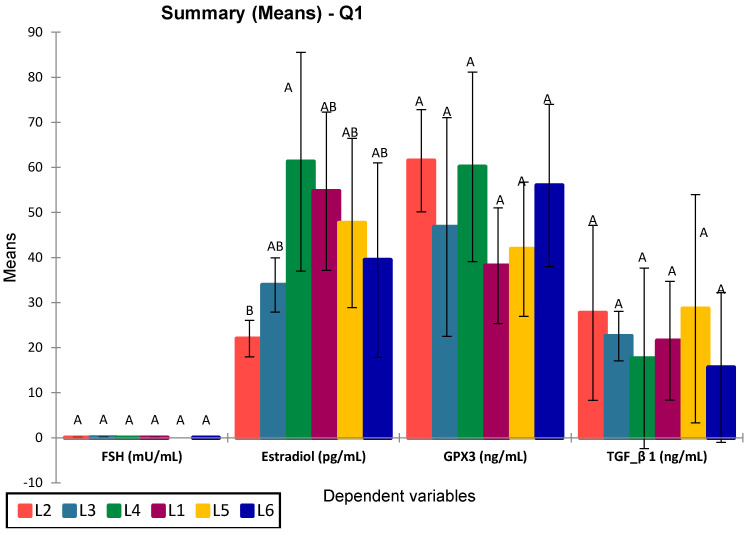
ANOVA for analyzed selected biomarkers in the study groups. Values sharing the same superscript are not statistically different (*p* > 0.05).

To assess the effects of treatments on serum biochemical parameters, data distribution and homogeneity of variances were first evaluated using the Shapiro–Wilk and Levene’s tests, respectively. Estradiol, GPX3, and TGF-β1 levels showed normal distribution across all experimental groups (Shapiro–Wilk *p* > 0.05) and homogeneity of variances (Levene’s test *p* > 0.05), justifying the use of one-way ANOVA. In contrast, as progesterone levels displayed non-normal distribution in groups L3–L5 (Shapiro–Wilk *p* = 0.031–0.043) and significant heteroscedasticity (Levene’s test *p* = 0.220; Bartlett’s test *p* < 0.001), a non-parametric approach was used. The Kruskal–Wallis test followed by Dunn’s post-hoc comparisons was applied to assess intergroup differences in progesterone concentrations. To evaluate the effects of treatments on hormonal and oxidative stress-related markers, a one-way ANOVA followed by Tukey’s post-hoc test was applied (Figure 11).

**Figure 12:**
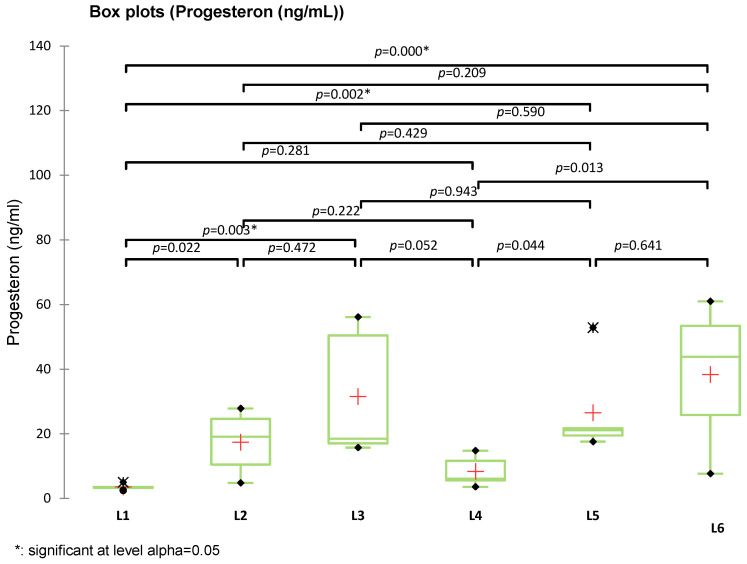
Box plot representation of serum progesterone levels (ng/mL) in experimental groups (L1–L6). Statistical comparisons were performed using Kruskal–Wallis, Tukey, and Dunnett tests (+: mean value of each group).

Among the analyzed parameters (FSH, estradiol, GPX3, and TGF-β1), only estradiol showed statistically significant differences between groups (*p* = 0.016), with significant differences between L4 vs. L2 (*p* = 0.015) and L3 vs. L1 (*p* = 0.025). Specifically, group L4 (treated with compound S) showed the highest estradiol levels, significantly greater than the untreated diabetic group (L2), with an apparent partial recovery in groups L5 and L6 (treated with sage extract and combined treatment, respectively). No significant differences were observed for FSH (*p* = 0.075), GPX3 (*p* = 0.212), or TGF-β1 (*p* = 0.821), although group L6 consistently showed elevated GPX3 and reduced TGF-β1 levels. The post-hoc Tukey (HSD) test for FSH at a 95% confidence interval found no statistically significant differences between groups (*p* > 0.05), The Dunnett test (two-sided) showed no significant FSH differences, with L5 closest to significance (*p* = 0.560). The graphical representation confirms the statistical results, with overlapping letters (A/B) denoting non-significant differences in Tukey’s grouping. Progesterone levels were significantly increased in all treated groups compared to the control group (L1), particularly in rats receiving metformin (L3, *p* = 0.003), sage extract (L5, *p* = 0.002), and the combination of sage and the synthetic compound (L6, *p* = 0.003). However, the differences between L3, L5, and L6 were not statistically significant (*p* = 0.590, 0.209, 0.641, respectively). The group treated with the synthetic compound alone (L4) did not differ significantly from the control (*p* = 0.281) but was significantly different from L6 (*p* = 0.013) (Figure 12).

**Figure 13:**
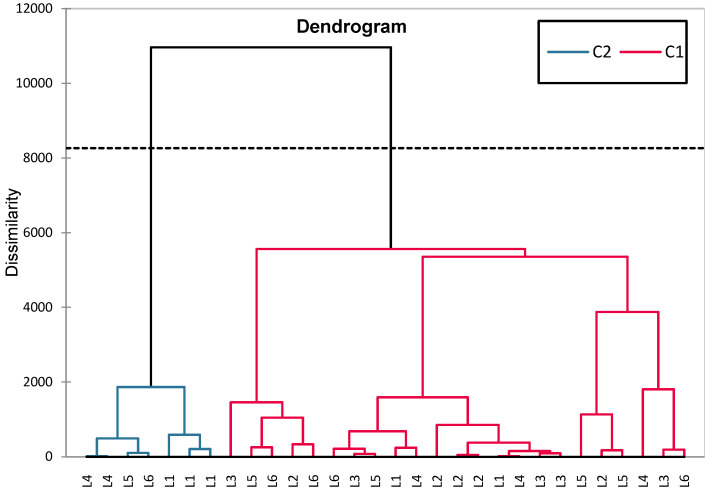
The HCA dendrogram constructed based on the set of variables FSH, Estradiol, Progesterone, GPX3, and TGF-β1.

The HCA (hierarchical clustering analysis) grouped L5 (sage) and L6 (synthetic compound + sage) together (C2, blue), suggesting a common sage effect. L4, L5, L6, and L1 (C2, red) were more homogeneous, while L2 (untreated) and L3 (metformin) clustered separately (C1, red), indicating higher variability. The clear separation of L2 confirms its distinct biological profile (Figure 13).

#### 3.4.4. Analysis of Relationships Between Variables—Statistical Correlations

To understand the relationships between hormonal markers and oxidative stress markers on one hand and blood glucose levels on the other, as well as between weight and glucose levels, correlation analyses were conducted. These analyses allow the identification of significant associations between variables and the determination of links between the effects of applied treatments and observed biological changes. For this purpose, Pearson/Spearman correlations were used, along with PCA analysis to assess the direction and intensity of relationships between the studied parameters, as well as multiple regression analyses using Xlstat 2023.3.0 (Basic+ version).

For biochemical markers, Pearson correlations were performed both on the entire dataset and separately for each group to identify general trends as well as specific changes in each lot (Table 5).

**Figure 14:**
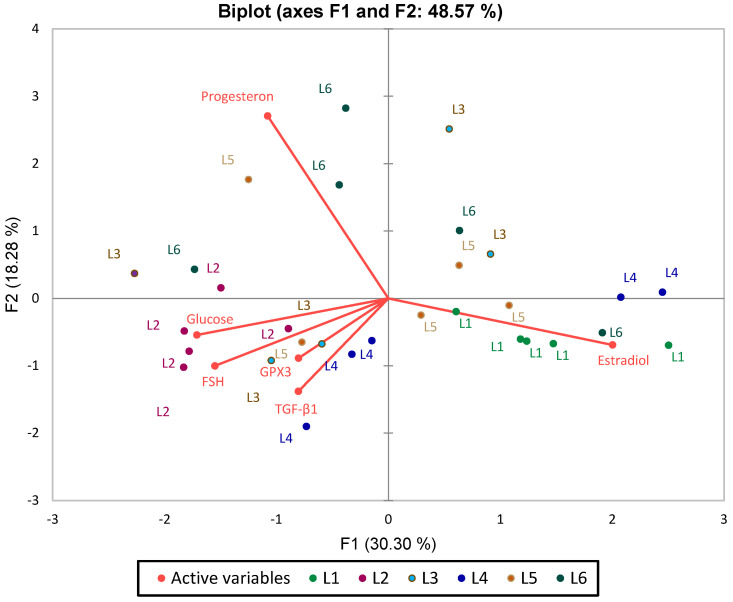
PCA Analysis Plot (Standardized Data) for Analyzed Biomarkers in Relation to Blood Glucose.

Principal Component Analysis (PCA) was applied as an exploratory tool to identify global patterns and similarities among experimental groups based on biochemical, oxidative, and hormonal parameters (Figure 14).

The PCA biplot analysis of the first two principal components (F1 and F2), which together explain 48.57% of the total variance, reveals key patterns in biomarker distribution and group differentiation. Blood glucose, FSH, and GPX3 emerge as the primary drivers of separation along the F1 axis, positioning the L2 group (untreated diabetes) distinctly to the left, highlighting its metabolic and oxidative imbalance. Conversely, estradiol is oriented toward the right, clustering with control (L1) and treated groups (L4, L5), indicating a strong association with hormonal modulation. TGF-β1, though contributing less to group separation, aligns with L4 and L5, and its proximity to GPX3 underscores the natural connection between inflammation and oxidative stress.

A clear reverse correlation between FSH and estradiol is observed along the F1 axis, consistent with physiological expectations. Additionally, blood glucose, progesterone, and GPX3 are positively correlated.

The L2 group is markedly distinct, characterized by elevated blood glucose, progesterone, and GPX3. In contrast, L4 and L6 show a strong association with estradiol, intended a pronounced effect of the synthetic compound and its combination with sage on this hormone. L3 (metformin treatment) is positioned opposite FSH.

PCA analysis confirms the results obtained from previous statistical tests, providing a clear perspective on the differences between groups. Data standardization allowed the elimination of scale differences between variables, ensuring a balanced representation of each parameter’s influence on the separation of experimental groups.

Regression analyses were exploratory, aiming to evaluate the potential of specific biomarkers to predict glycemic status or hormonal levels. Glucose concentration was introduced as the independent variable, while FSH, estradiol, progesterone, GPX3, and TGF-β1 were analyzed as dependent variables. The results are summarized in Table 6. Given the multifactorial nature of endocrine–metabolic interactions in diabetes, this analysis aimed to identify potential predictors among the measured biomarkers.

The results, summarized in Table 6, indicate that blood glucose level was not a significant predictor for FSH, estradiol, progesterone, or TGF-β1 levels (all *p* > 0.05). However, a statistically significant model was obtained for GPX3, where glucose levels explained approximately 24% of the variance (R^2^ = 0.239, *p* = 0.01), suggesting a moderate association between glycemic status and antioxidant enzyme activity.

The analysis of the regression models applied to the dataset revealed relatively weak relationships between the studied biomarkers, without identifying blood glucose level as a clear predictor for the dependent variables, except GPX3.

## 4. Discussions

In vivo experimental models have provided essential insights into the pathophysiological mechanisms involved in diabetes mellitus, facilitating the investigation of endocrine and metabolic changes associated with the disease and the testing of potential therapies [18,26,29].

Within the current study, we employed a model of alloxan-induced diabetes in female Wistar rats to investigate the impact of chronic hyperglycemia on physiological parameters, hormonal status, and oxidative stress markers with a particular focus on its impact on the female reproductive system. The effects of metformin, a synthetic sulfonamide, and sage extract were tested individually and in combination, with a control group serving as a baseline to evaluate diabetes-induced imbalances and treatment effects. The study was conducted in accordance with ethical principles for animal research, complying with all international and national regulations.

Prior to the in vivo study, in vitro analyses of antioxidant capacity were conducted for both the synthesized sulfonamide compound and the *Salvia officinalis* extract synthetic compound exhibited moderate antioxidant activity in the DPPH test (IC_50_ = 0.018 mg/mL), and no activity according to the FRAP method. In contrast, the sage extract demonstrated high antioxidant activity across all methods used (DPPH, ABTS, and FRAP). Phytochemical analyses of the *Salvia officinalis* extract revealed a high content of polyphenols (30.12 ± 6.19 g% tannic acid), phenolic acids (26.88 ± 1.06 g% chlorogenic acid), and flavonoids (8.33 ± 1.53 g% rutin), indicating a direct correlation between the extract’s chemical composition and its biological activity. UHPLC-HRMS analysis confirmed the presence of 54 polyphenolic bioactive compounds, including phenolic acids (rosmarinic acid, salvianolic acid, ferulic acid, chlorogenic acid) and flavonoids (luteolin, quercetin, kaempferol, catechin, and glycitein), known for their antioxidant and anti-inflammatory potential [45].

Within the in vivo experiment, the alloxan-induced diabetes model generated consistent severe hyperglycemia in the rats in the third week in experimental groups L2–L6) (*p* < 0.01 compared to the control group), confirming β-cell destruction by oxidative stress.

Diabetes-induced hyperglycemia was accompanied by progressive weight loss, particularly in the untreated group (L2), and the S-treated group (L4), reflecting a metabolic imbalance. This weight loss in alloxan-induced diabetic rats has been reported in similar studies [46]. The groups treated with *Salvia officinalis* extract (L5) and the combination therapy (L6) showed a moderate reduction in body weight, compared to groups L3 and L4, suggesting a potential protective role. These findings are consistent with previous reports indicating the positive metabolic effects of sage extracts, possibly through their antioxidant and anti-inflammatory properties [47]. Based on weekly blood glucose measurements and data analysis the hypoglycemic efficacy of the applied treatments showed significant variation. The observed variability in blood glucose levels, particularly in groups L4–L6 during weeks 4–8, may reflect individual differences in glycemic response due to intrinsic metabolic heterogeneity, differences in alloxan sensitivity [29], and oral absorption of treatments. Incomplete gastric retention of orally administered substances may also result in variable rates of absorption, affecting study outcomes [45]. Despite the standardization of administration and handling, inter-individual stress responses may have further contributed to glycemic variability.

Following the treatments, blood glucose variation was significantly different among groups L2, L3, L4, and L6 (*p* < 0.05), highlighting different responses. Metformin (L3) displayed efficacy in reducing blood glucose, confirming its beneficial effects on glucose metabolism. There was considerable individual variability within the group, as reflected by the large range (72–318 mg/dL) and Levene’s test (*p* < 0.05).

The hypoglycemic effect of metformin (group L3) by increasing insulin sensitivity and reducing hepatic glucose production is well known as one of the most commonly used treatments for T2DM. Regarding the animal models, our study is consistent with previous research [48], which reported a significant reduction in blood glucose levels and high variability in alloxan-induced diabetic rats treated with metformin (*p* < 0.05).

The synthetic sulfonamide S used in group L4 had moderate efficacy in lowering blood glucose but led to significant weight loss with significant individual variability observed within the group (Levene’s test *p* < 0.05).

The sulfonamide moiety (–SO_2_N-) is a pharmacologically active group that exhibits a wide range of biological activities, including insulin-releasing antidiabetic effects. In a study evaluating several novel sulfonamide derivatives for their hypoglycemic activity in alloxan-induced diabetic rats, the authors demonstrated that these compounds exhibited hypoglycemic effects, comparable to those of the sitagliptin used as a reference drug [49]. The study by Markowicz-Piasecka et al., which evaluated the effect of a series of novel sulfonamides on endothelial cells, highlighted a glucose utilization rate comparable to the control (metformin) [50]. These findings reinforce the potential of sulfonamides as promising antidiabetic agents, a hypothesis further supported by our study.

The sage extract used in group L5 exhibited weak effects on blood glucose but had a moderate protective impact on body weight. In group L5, the differences were not significant compared to the untreated group (L2) (*p* = 0.401). However, in group 6, the combination of the synthetic compound with *Salvia officinalis* extract (L6) led to a glycemic reduction comparable to that of metformin (L3), with final glucose levels at week 8 reaching 121 mg/dL and 112 mg/dL, respectively. While the statistical comparison between L6 and L3 at week 8 did not reach significance (*p* = 0.0070), the pairwise comparison from week 5 to 7 showed a significant difference between L6 and L3 (*p* = 0.0016–0.002).

Previous studies have demonstrated the anti-diabetic potential of *Salvia officinalis*; however, the results remain inconsistent, suggesting a distinct mechanism of action compared to conventional antidiabetic drugs. Alarcon-Aguilar et al. (2002) demonstrated that after an intraperitoneal injection of a water-ethanolic sage extract, blood glucose levels significantly decreased in fasted normal mice and mildly alloxan-diabetic mice, but no significant reduction was observed in severely alloxan-diabetic mice [51]. Additionally, Eidi et al. (2005) reported that after an intraperitoneal injection of an alcoholic sage extract, blood glucose levels significantly decreased in fasted streptozotocin-diabetic rats but the effect was not associated with an increased release of insulin [52]. In the study by Lima et al., sage was administered as a tea for 14 days, resulting in a significant reduction in fasting plasma glucose levels from 8.8 mM to 6.8 mM (*p* ≤ 0.01) in normal mice. However, during the intraperitoneal glucose tolerance test (ipGTT), no improvement in glucose clearance was observed compared to the control group [53]. The differences observed compared with our study, highlight the impact of administration method, diabetes model, and duration of treatment on sage’s hypoglycemic potential.

Our results confirm studies indicating that combined therapies using pharmaceutical compounds and natural products have effects superior to monotherapy, improving both glycemic control and diabetes complication prevention [32,33]. Statistical analysis using the Aligned Rank Transform (ART) ANOVA was used to investigate the independent and combined effects of the synthetic compound (S) and sage extract on glucose levels during the treatment period (weeks 4 to 8). The results revealed statistically significant main effects for both S (F = 10.10, *p* = 0.0022) and sage (F = 15.80, *p* = 0.0002), indicating that each treatment contributed independently to reducing glucose concentrations. However, the interaction term between S and sage (F = 0.63, *p* = 0.431) was not statistically significant, suggesting no synergistic effect. These results support the hypothesis that the treatments act through complementary mechanisms.

A series of biochemical parameters were measured in the plasma samples of the animals to evaluate the influence of diabetes on oxidative stress and reproductive system function.

Statistical analysis highlighted significant differences between the studied groups, particularly for estradiol and progesterone, confirming the impact of diabetes on hormonal homeostasis.

For FSH, even though ANOVA did not show significant differences (*p* = 0.075) between the groups. Dunnett’s test showed significant differences in group 3 (0 = 0.030).

Although FSH levels did not differ significantly between groups (*p* = 0.075) the mean values in the untreated group showed a notable increase. As diabetes disrupts ovarian function, leading to a decrease in estradiol levels and, consequently, a compensatory increase in FSH through hypothalamic-pituitary negative feedback [9].

On the other hand, an increase in FSH was also observed in the metformin-treated group, whereas in groups L4–L6, a decrease in FSH levels was noted compared to L1. It is known that metformin can improve ovarian sensitivity to insulin but can also cause a temporary increase in gonadotropins through indirect mechanisms [26,48.]. For estradiol, ANOVA (*p* = 0.016) indicated significant differences between groups. Induced diabetes significantly reduced estradiol levels (L2 vs. L1, *p* = 0.025, Dunnett test), whereas treatments with the synthetic compound (L4, *p* = 0.002, Tukey test) and the combination of sage + synthetic compound (L6, *p* = 0.008, Dunnett test) proved a protective effect of treatments on estradiol levels possible due to antioxidant and estrogenic activity [54]

Experimental models of diabetes have been associated with estradiol suppression, as demonstrated by Ramalho-Santos et al. (2008) in animal models [55]. Their study indicated that diabetes is linked to decreased estrogen synthesis due to alterations in the hypothalamic-pituitary-ovarian axis, increased oxidative stress, and mitochondrial dysfunction [55]. In the group treated with metformin, a positive effect on estradiol levels was observed, although it was not sufficient to fully restore hormonal levels to those of the control group (*p* > 0.05). Treatments with the synthetic compound (L4) and sage (L5, L6) led to an increase in estradiol, suggesting a protective effect on ovarian function, similar to findings in studies investigating antioxidant and hormone-regulating treatments [33,47]. For progesterone, the differences between groups were significant (Kruskal-Wallis *p* = 0.0021). Progesterone levels were significantly increased in groups treated with metformin (L3), *Salvia officinalis* extract (L5), and combination therapy (L6), compared to the healthy control group (L1). Among these, metformin elicited the highest progesterone response (*p* = 0.002 vs. L1), followed closely by the combination (*p* = 0.013) and sage extract alone (*p* = 0.044). Treatment with the synthetic compound alone (L4) did not result in a significant change in progesterone levels compared to the healthy control group (L1, *p* = 0.281). Confirming the obtained results, some studies suggest that diabetes may lead to hormonal imbalances by affecting luteal secretion and insulin sensitivity, which can increase progesterone levels [22,25]. Other research indicates that progesterone levels may decrease in advanced diabetes due to ovarian dysfunction and dysregulation of the hypothalamic-pituitary-ovarian axis and shows that the influence of progesterone on glucose metabolism is bidirectional: increased progesterone can induce insulin resistance, worsening diabetes, while diabetes, in turn, can alter progesterone secretion [56].

The correlation analysis highlighted relationships between the hormonal axis, and blood glucose, suggesting distinct effects of diabetes and treatments on endocrine homeostasis. A consistent finding was the negative correlation between FSH and estradiol. In the healthy control group (L1), this correlation was moderate (r = −0.362, *p* = 0.049), reflecting normal physiological mechanisms where an increase in FSH is associated with a decrease in estradiol and vice versa. In contrast, in the untreated diabetic group (L2), the relationship became abnormally rigid (r = −0.996, *p* = 0.008), suggesting an endocrine imbalance induced by diabetes, likely due to its influence on the regulation of the hypothalamic-pituitary-gonadal axis, making the feedback mechanisms less flexible [57]. A correlation of −0.996 suggests that whenever one variable changes, the other follows in a nearly absolute, linear manner, which is unusual for biological systems that typically exhibit variability showing a dynamic and adaptive character rather than rigidly fixed [57]. Progesterone level was correlated to glucose level (r = 0.374, *p* = 0.042) confirming that increased glycemia may affect luteal hormone dynamics.

Metformin treatment (L3) appeared to have a specific effect on FSH regulation, as in this group, blood glucose correlated negatively with FSH (r = −0.904, *p* = 0.035).

Overall, the results indicate that induced diabetes significantly affects sex hormones, particularly estradiol, confirming literature findings that support the impact of diabetes on the female reproductive system [22,56].

GPX3 (glutathione peroxidase 3) and TGF-β1 (transforming growth factor-beta 1) were selected as biomarkers related to oxidative stress regulation and inflammation, both of which are closely linked to metabolic dysfunction and diabetes progression [35]. GPX3 showed wide variation in the experimental groups, ranging from 5.51 ng/mL to 49.83 ng/mL, with a mean of 38.54 ± 29.64 ng/mL; for TGF-β1, concentrations also varied widely, ranging from 0.32 ng/mL to 57.33 ng/mL, with a mean value of 35.6 ± 29.64 ng/mL.

Statistical analysis revealed no significant intergroup differences for GPX3 levels (ANOVA *p* = 0.212), nor for TGF-β1, for which all applied ANOVA tests yielded non-significant results, indicating that this factor is not strongly influenced by diabetes or by the administered treatments in the study groups.

GPX3 levels correlated positively with blood glucose (r = 0.440, *p* = 0.015) suggesting that high glycemia may induce a compensatory upregulation of antioxidant defense. GPX3 was also negatively correlated with FSH and estradiol (*p* = 0.039), indicating an interaction between oxidative stress and hormonal imbalances. These observations suggest that oxidative stress is closely linked to hormonal imbalances in diabetes, potentially contributing to observed endocrine dysfunctions [10,21,58]. Studies indicate that diabetic patients with chronic inflammation and high oxidative stress generally exhibit low GPX3 levels, as the body cannot efficiently compensate for oxidative stress [58]. However, experimental studies on animals and humans report that in the early phase of diabetes, GPX3 may initially increase as a compensatory mechanism against rising reactive oxygen species (ROS), but as oxidative stress becomes chronic, this mechanism depletes, and GPX3 levels decline [58].

Notable, the group treated with sage + synthetic compound (L6) exhibited a significant positive correlation between GPX3 and FSH (r = 0.929, *p* = 0.023), which may indicate an indirect effect of the treatment on hormonal regulation through mechanisms related to oxidative stress. The active compounds of the sage extract, particularly salvianolic acids, activate AMPK signaling, improve mitochondrial function, and reduce oxidative damage [52]. In contrast, the group treated only with sage (L5) did not show significant correlations between the analyzed biomarkers.

Regression analysis revealed that blood glucose was the significant predictor only for GPX3 levels (R^2^ = 0.239, *p* = 0.010). No significant relations were identified for hormonal parameters, suggesting that their regulation is influenced by additional, possibly nonlinear, or multifactorial mechanisms. These findings highlight the connection between glycemic imbalance and oxidative stress, as well as the complexity of endocrine responses in the diabetic context.

The observed glycemic improvements following the combined treatment may hold translational relevance for female reproductive health. Rodent models remain highly relevant for investigating reproductive endocrine function, as their ovarian cycle exhibits periodic fluctuations in estrogen and progesterone, closely mirroring human hormonal dynamics [59]. The presence of steroid hormone receptors in target reproductive tissues such as the uterus and mammary glands enables translational insights into hormone-mediated developmental processes [60]. However, extrapolating findings from rodent models to human physiology presents important limitations. Despite substantial physiological, and metabolic similarities between rodents and humans, differences in anatomy, morphogenesis, duration, and regulation of the estrous versus menstrual cycle, oocyte release patterns, and lifespan must be acknowledged when translating findings to the clinical context [60]. Moreover, environmental factors such as light cycles and stress have a greater impact on rodent reproductive behavior, limiting the predictability of outcomes [61]. Further studies will be required to assess the therapeutic potential of this combination strategy in the context of fertility preservation or endocrine metabolic regulation in diabetic women.

A limitation of the present study is the absence of histopathological data, which are commonly used to confirm and stage tissue-level alterations in diabetic models. However, detailed morphological and histological findings from the same experimental cohort are being addressed in a separate publication focusing specifically on structural and cellular changes induced by diabetes and treatment.

## 5. Conclusions

This study demonstrates that chronic hyperglycemia induced by alloxan significantly disrupts endocrine and metabolic homeostasis in female rats, which is consistent with the scientific literature. Treatments with metformin, a synthetic sulfonamide (S), and *Salvia officinalis* extract—administered alone or in combination with S—revealed differential efficacy across metabolic and hormonal parameters.

Diabetes-induced estradiol suppression and FSH elevation were observed, consistent with hypothalamic-pituitary-gonadal axis dysregulation. Oxidative stress, evaluated via GPX3, was the only biomarker significantly predicted by blood glucose levels (R^2^ = 0.239, *p* = 0.010), highlighting the link between hyperglycemia and redox imbalance.

Among the interventions, the combination of the synthetic compound with sage (L6) proved the most effective in reducing blood glucose levels, comparable to metformin by the end of the experiment. However, synergic action wasn’t demonstrated by statistical analysis. The combination therapy also contributed to the stabilization of body weight and showed favorable hormonal modulation confirmed by a significant positive correlation between GPX3 and FSH, pointing to a possible interaction between antioxidant regulation and endocrine signaling.

The findings support the relevance of combining alternative and standard treatments to optimize glycemic control and regulate hormonal homeostasis.

## Figures and Tables

**Table 1:** Concentrations of relevant active principles and antioxidant activity values.

Vegetal Extract	Total Polyphenols (g% Tannic Acid)	Phenolic Acids (g% Chlorogenic Acid)	Total Flavonoids (g% Rutozide)
*Salvia officinalis*	30.12 (±6.19)	26.88 (±1.06)	8.33 (±1.53)
	**DPPH** **IC_50_ (mg/mL)**	**ABTS** **IC_50_ (mg/mL)**	**FRAP** **EC_50_ (mg/mL)**
*Salvia officinalis*	0.123	0.0356	0.0543

**Table 2:** Correlation coefficient between the methods used and between antioxidant activity and polyphenolic compound content.

Correlation	r	R^2^
ABTS vs. DPPH	1.00	0.9900
ABTS vs. FRAP	−0.999	0.9293
DPPH vs. FRAP	−0.998	0.9643
Total polyphenols vs. IC_50_ (DPPH, ABTS)	−0.265	
Phenolic acids vs. IC_50_ (DPPH, ABTS)	0.991	
Total flavonoids vs. IC_50_ (DPPH, ABTS)	0.468	

DPPH: 2,2-Diphenyl-1-picrylhydrazine; ABTS: 2,2′-Azino-bis(3-ethylbenzothiazoline-6-sulfonic acid); FRAP: Ferric Reducing Antioxidant Power.

**Table 3:** Quantitative analysis results for bioactive compounds in sage extract.

Compound	Chemical Formula	Monitored Ion [M-H]^−^	Retention Time (min)	Amount (mg/g Extract)
Catechin	C_15_H_14_O_6_	289.0718	5.48	5.96
Luteolin	C_15_H_10_O_6_	285.0405	11.82	5.01
Kaempferol	C_15_H_10_O_6_	285.0405	15.88	31.2
Quercetin	C_15_H_10_O_7_	301.0354	10.33	3.12
Hesperidin	C_16_H_14_O_6_	301.0718	15.53	3.78
Rutin	C_27_H_30_O_16_	609.1461	8.44	5.12
Pinocembrin	C_15_H_12_O_4_	255.0663	18.31	3.12
Galangin	C_15_H_10_O_5_	269.0456	16.67	0.74
Apigenin	C_15_H_10_O_5_	269.0450	13.22	1.95
Gallic acid	C_7_H_6_O_5_	169.0143	1.18	1.41
Rosmarinic acid	C_18_H_16_O_8_	359.0773	13.38	37.85
Ferulic acid	C_10_H_10_O_4_	193.0507	19.3	3.58
Salvianolic acid A	C_26_H_22_O_10_	493.1140	14.95	6.27
Caffeic acid	C_9_H_8_O_4_	179.0350	6.44	1.46
Cinnamic acid	C_9_H_8_O_2_	147.0452	2.14	1.96
*p*-Coumaric acid	C_9_H_8_O_3_	163.0395	8.85	0.64
Glycitein	C_16_H_12_O_5_	283.0612	19.56	18.40
Chlorogenic acid	C_16_H_18_O_9_	353.0878	6.08	6.98
Pinostrobin	C_16_H_14_O_4_	269.0820	18.46	2.86
Vanillic acid	C_8_H_8_O_4_	167.0350	2.36	11.98
Syringic acid	C_9_H_10_O_5_	197.0456	6.24	0.51
Ellagic acid	C_14_H_6_O_8_	300.9990	12.04	0.56
Abscisic acid	C_15_H_20_O_4_	263.1289	14.79	0.41
Naringenin	C_15_H_12_O_5_	271.0612	15.51	1.81

**Table 4:** Summary of biochemical parameter analysis results, expressed as mean values and standard deviation per group.

Lot	FSH (mU/mL)	LH (mUI/mL)	Estradiol (pg/mL)	Progesteron (ng/mL)	GPX3 (ng/mL)	TGF-β1 (ng/mL)
L1	0.04 ± 0.05	<0.07 *	54.74 ± 17.5	3.59 ± 0.90	38.20 ± 12.84	21.55 ± 13.15
L2	0.05 ± 0.03	<0.07	22.00 ± 4.1	17.38 ± 9.6	61.50 ± 11.3	27.73 ± 19.4
L3	0.09 ± 0.09	<0.07	33.92 ± 6.0	31.56 ± 19.9	46.78 ± 24.2	22.57 ± 5.5
L4	0.00 ± 0.0	<0.07	61.29 ± 24.2	8.34 ± 4.6	60.13 ± 21.0	17.65 ± 20.0
L5	0.00 ± 0.0	<0.07	47.69 ± 18.7	26.55 ± 14.8	41.88 ± 14.9	28.66 ± 25.3
L6	0.02 ± 0.03	<0.07	39.44 ± 21.5	38.35 ± 21.6	56.01 ± 18.0	15.60 ± 16.6

* Limit of quantification for the test.

**Table 5:** Significant correlation test results (expressed as *p*-values) by groups.

Group	Pearson Correlations
L1	FSH vs. Estradiol (ρ = −0.362, *p* = 0.049), significant negative correlation Blood glucose vs. GPX3 (ρ = −0.956, *p* = 0.011), significant negative correlation
L2	FSH vs. estradiol (ρ = −0.996, *p* = 0.008), significant negative correlation GPX3 vs. FSH (ρ = −0.897, *p* = 0.039), significant negative correlation GPX3 vs. estradiol (ρ = −0.923, *p* = 0.025), significant negative correlation
L3	Blood glucose vs. FSH (ρ = −0.904, *p* = 0.035), significant positive correlation
L4	Estradiol vs. progesteron (ρ = 0.979, *p* = 0.004), significant positive correlation GPX3 vs. TGF-β (ρ = 0.938, *p* = 0.018), significant positive correlation
L5	No significant correlations between biomarkers
L6	GPX3 vs. FSH (ρ = 0.929, *p* = 0.023), significant positive correlation

**Table 6:** Regression analysis of biomarkers: R^2^, F-statistic, and significance levels.

Parameter	R^2^	F-Statistic	*p*-Value (Pr > F)	Conclusion
FSH	0.046	1.206	0.286	Not significant
Estradiol	0.048	1.186	0.6292	Not significant
Progesterone	0.059	1.580	0.220	Not significant
GPX3	0.239	7.840	0.010	Significant
TGF-β1	0.0369	0.925	0.345	Not significant

## Data Availability

The original contributions presented in this study are included in the article/Supplementary Materials.

## Supplementary Materials

The following supporting information can be downloaded at: https://www.mdpi.com/article/10.3390/biomedicines13040922/s1, Figure S1. Calibration curves for sage extract and vitamin C using DPPH method; Figure S2. Calibration curve for sage extract using ABTS method; Figure S3. Calibration curve for sage extract using FRAP method; Figure S4. Calibration curve for the determination of IC 50 for synthetic sulphonamide S; Figure S5. The LC-HRMS chromatogram for the sage extract identified the following compounds: salvianolic acid A (*m*/*z* 493.11401, TR 14.95), rosmarinic acid (*m*/*z* 359.0772, TR 13.38), rosmanol (*m*/*z* 345.17077, TR 17.15), carnosic acid (*m*/*z* 331.1915, TR 23.44), carnosol (*m*/*z* 329.17585, TR 21.00), azelaic acid (*m*/*z* 187.0976, TR 13.99), and ursolic acid (*m*/*z* 455.3530, TR 25.26). The chromatograms were extracted from TIC (Total Ion Chromatogram) using a 5 ppm window; Figure S6. Full MS and MS-MS chromatogram for the sage extract, where rosmanol was identified; Figure S7. Full MS and MS-MS chromatogram for the sage extract, where rosmanol was identified. From top to bottom: TIC (Total Ion Chromatogram) showing the identified compound at 17.15 min, full MS chromatogram for the extracted mass *m*/*z* 345.17, MS-MS chromatogram for the precursor ion with the mentioned mass, and the characteristic ionic fragments *m*/*z* 283.17, 268.14, and 227.10 (TR 7.14), identified as rosmanol, according to MassBank. The chromatograms were extracted from TIC using a 5 ppm window; Table S1. Chemical compounds qualitatively identified by UHPLC-HRMS/MS; Table S2. Effect of metformin, the synthetic compound, and sage on body weight (G) of rats with induced diabetes at different treatment time points. Data are presented as median values per group and time point, due to the non-normal distribution of the variable; Table S3. Effect of metformin, the synthetic compound, and sage on blood glucose levels in rats with induced diabetes at different treatment time points. Data are presented as median values per group and time point, due to the non-normal distribution of the variable; Table S4. Summary of pairwise comparisons of blood glucose level between treatment groups over time using Kruskal–Wallis test followed by Dunn’s post-hoc analysis with Bonferroni correction (α = 0.0033).
