# Supplementary material for: Multilevel Assessment of Glycemic, Hormonal, and Oxidative Parameters in an Experimental Diabetic Female Rat Model
Source: Biomedicines. 2025 Apr 9;13(4):922. doi: 10.3390/biomedicines13040922 (PMC12024543; doi:10.3390/biomedicines13040922)
Supplement: Supplementary file 1 [file biomedicines-13-00922-s001.zip › biomedicines-3551103-supplementary.pdf]

Supplementary Materials

Materials and methods

Analysis of sage extract and its antioxidant activity

The analytical methods used for determining phenolic and flavonoid compounds are based on colorimetric reactions and spectrophotometry, utilizing specific chemical principles for each type of analyzed compound, according to literature (Costea et al., 2022)

Determination of total flavonoid content (TF) was based on the reaction of flavonoids with $AlCl_3$ to form a stable complex measurable by spectrophotometry. Dry *Salvia Officinalis* extract (0.2 g) were dissolved in 50% ethanol. Volumes of 0.4 mL, 0.6 mL, 0.8 mL, 1 mL, and 1.2 mL were poured into 10 mL volumetric flasks and then reacted with sodium acetate solution (100 g/L) and aluminum chloride (25 g/L), followed by dilution to 10 mL. After 45 min, absorbance is measured at 427 nm after 45 minutes (Jasco V-530 spectrophotometer, Tokyo, Japan), using rutin as a calibration standard. Results are expressed in mg rutin equivalents per gram of sample.

Determination of total phenolic acid content (TPA) relies on the formation of nitro derivatives with nitrous acids. Dry extract (0.2 g) was dissolved in ethanol 50%. Volumes of 0.8 mL, 1 mL, 1.2 mL, 1.4 mL, and 1.6 mL were poured into 10 mL volumetric flasks. Hydrochloric acid (0.5 M), Arnow reagent, and sodium hydroxide (85 g/L) were successively added before adjusting the volume to 10 mL. Absorbance is measured immediately at 525 nm, with chlorogenic acid used as a calibration standard. Results are expressed in mg chlorogenic acid equivalents per gram of sample.

Determination of total phenolic content (TP) used the Folin–Ciocalteu method. Extract was dissolved in ethanol 50%. Volumes of 0.5 mL, 0.6 mL, 0.7 mL, 0.8 mL, and 0.9 mL were poured into 10 mL volumetric flasks and adjusted to 1 mL by adding distilled water. Samples react with Folin–Ciocalteu's phenol reagent and sodium carbonate solution (200 g/L). After 40 minutes in dark conditions, absorbance is measured at 725 nm. Tannic acid serves as the standard, and results are expressed in mg tannic acid equivalents per gram of sample.

Determination of Antioxidant Activity

The free radical scavenging activity of the plant extract was assessed using the DPPH (2,2-diphenyl-1-picrylhydrazyl) assay. Equal amounts (0.25 g) of dry extract were dissolved in 50% ethanol. Aliquots ranging from 10 to 100 µL were diluted to 10 mL with the same solvent and mixed with 0.5 mL of the diluted extract solution and 3 mL of 0.1 mM DPPH solution (Sigma-Aldrich, Germany). The reaction mixtures were incubated in the dark for 30 minutes, and absorbance was measured at 515 nm using a Jasco spectrophotometer (Japan). Ascorbic acid (Sigma-Aldrich, Germany) was used for calibration in the 2–22 µg/mL concentration range.

DPPH inhibition (%) was calculated using the formula: *% Inhibition = [(A blank – A sample)/A blank]x100,* where A is absorbance

IC_{50} values (mg/mL) were determined from inhibition curves, representing the extract concentration required to scavenge 50% of DPPH radicals (Figure S1).

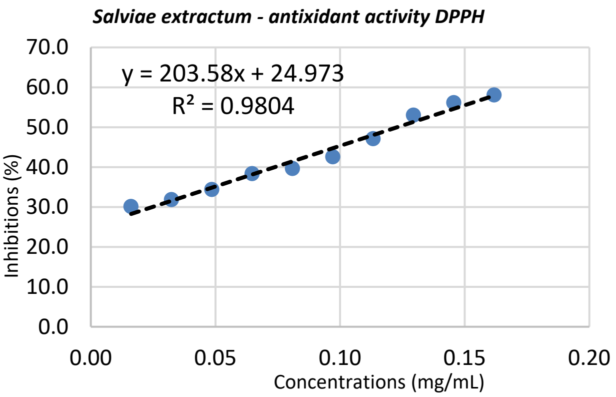

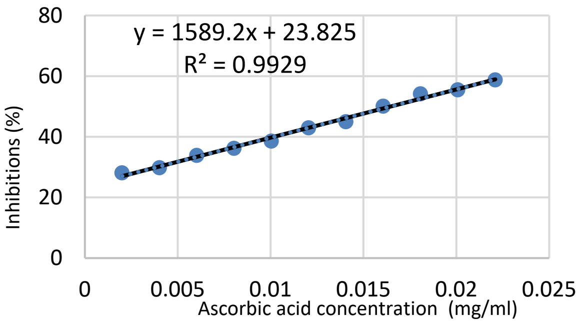

Figure S1. Calibration curves for sage extract and vitamin C using DPPH method

The ABTS assay was also conducted. ABTS (7.4 mM) reagent was reacted with potassium persulfate (2.6 mM, $K_2S_2O_8$) and incubated in the dark at room temperature for 16 hours before use. About 0.25 g of dry extract was dissolved in 50% ethanol. Aliquots ranging from 10 to 100 μL were diluted to 10 mL with the same solvent. Then, 0.5 mL of the diluted extract solution was mixed with 3 mL of ABTS•+ solution (diluted in ethanol).The mixtures were stirred and incubated in the dark for 6 minutes. Absorbance was measured at 734 nm with absolute ethanol as the blank.

ABTS•+ inhibition (%) was determined using the formula: *%inhibitions = [(A (t=0 min) – A (t =6 min))/A (t-o min)] x100*

A (t=0 min) = absorbance of the blank sample (ABTS•+ solution in the absence of tested compounds);

A (t=6 min) = absorbance of the vegetal extract (ABTS•+ solution in the presence of tested compounds).

The IC_{50} value, was determined from the plotted graph of radical scavenging activity (%) versus extract concentration (mg/mL) (Figure S2. A lower IC_{50} value indicates higher antioxidant activity, as it signifies that a smaller concentration of the extract is needed to achieve significant free radical scavenging.

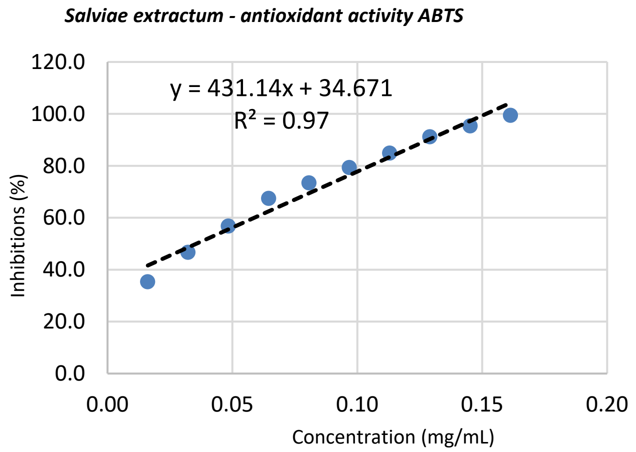

Figure S2. Calibration curve for sage extract using ABTS method

The ferric reducing power of plant extracts was evaluated using a modified FRAP assay, which measures the antioxidant potential by the reduction of ferric iron (Fe^{3+}) to ferrous iron (Fe^{2+}), resulting in a blue color (Costea et al., 2022).

An amount of 0.25 g dry extract were dissolved in 50% ethanol. Aliquots (50–500 μL) were diluted to 10 mL, mixed with phosphate buffer (pH 6.6) and $K_3(FeCN)_6$ (1%), then heated at 50°C for 20 minutes. Trichloroacetic acid was added, followed by distilled water and $FeCl_3$ (0.1%), and left to stand for 10 minutes. Absorbance was recorded at 700 nm, with a blank sample containing $FeCl_3$ (0.1%) and distilled water. The EC50 value (mg/mL), representing the effective concentration required to achieve 50% of the maximal antioxidant effect (absorbance = 0.5), was determined from the absorbance-concentration curve (Figure S3).

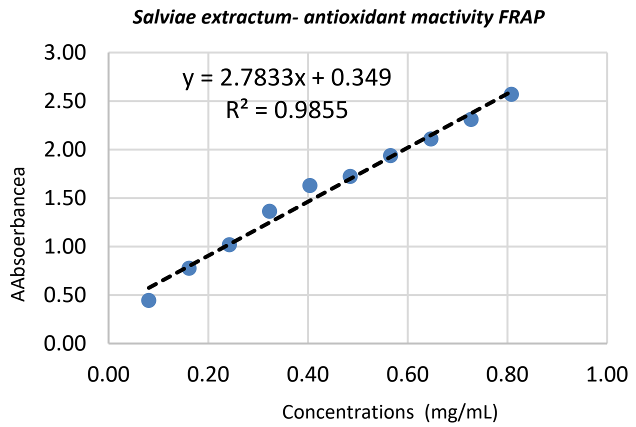

Figure S3. Calibration curve for sage extract using FRAPmethod

For the synthetic sulphonamide IC_{50} was determined by DPPH method, according to described procedure (Figure S4)

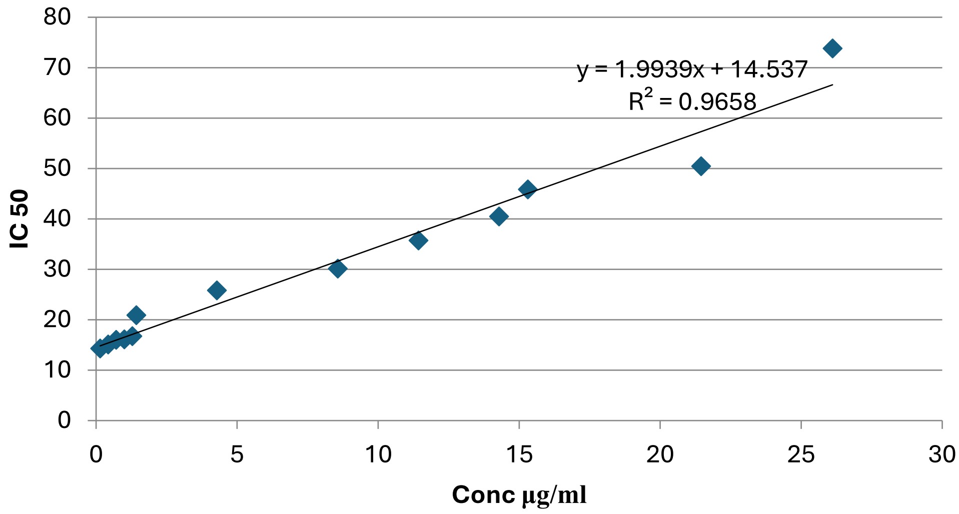

Figure S4. Calibration curve for the determination of IC 50 for synthetic sulphonamide S

Bioanalytical methods (according to the producer protocol)

Determination of TGF-β1 by ELISA Immunoassay (Enzyme-Linked Immunosorbent Assay)

The method used for TGF-β1 determination is based on an immune-enzymatic reaction, where specific antibodies for TGF-β1 are immobilized on microplates. Plasma samples, separated by centrifugation and diluted 1:10 with a specific buffer, were distributed into the individual wells of the microplate and incubated to allow the binding of TGF-β1 present in the samples to the capture antibodies. After multiple washing steps to remove unbound molecules, a secondary biotinylated antibody was added, which binds to the formed antigen-antibody complex. This complex was detected by adding a streptavidin-HRP (horseradish peroxidase) conjugate, which binds to biotin. In the presence of the TMB (tetramethylbenzidine) substrate, HRP catalyzes a colorimetric reaction, where the intensity of the developed color is directly proportional to the TGF-β1 concentration in the sample. The reaction was stopped using a diluted sulfuric acid solution, and absorbance was measured at 450 nm. A standard curve was generated from the serial dilutions of standard solutions, and the TGF-β1 concentration in unknown samples was determined by interpolating absorbance values on the standard curve.

Detection limit: 8.6 pg/mL, enabling precise measurement of low TGF-β1 concentrations. No cross-reactivity with other similar cytokines was observed.

Determination of GPX3 by Sandwich ELISA Immunoassay

Plasma samples (diluted 1:200) were added to the microplate wells, where GPX3 from the samples binds to specific polyclonal antibodies immobilized on the plate surface. After washing unbound compounds, a second biotinylated antibody was added, which specifically binds to GPX3. Detection was performed using a streptavidin-peroxidase (HRP) conjugate, which, in the presence of TMB (tetramethylbenzidine) substrate, catalyzes a colorimetric reaction. The reaction was stopped by adding 100 µL of stop solution (diluted sulfuric acid). The color intensity is proportional to the GPX3 concentration in the sample and was measured at 450 nm. Standard GPX3 solutions were serially diluted to generate a standard curve ranging from 0.5 ng/mL to 32 ng/mL, and the concentrations of the unknown samples were determined by interpolation.

Detection limit: 100 pg/mL. The method exclusively detects GPX3, with no cross-reactivity with other GPX isoforms (such as GPX1, GPX2, or GPX4).

Determination of Hormones (FSH, Estradiol, LH, and Progesterone)

FSH, estradiol, LH, and progesterone levels in serum were determined using a direct chemiluminescence-based sandwich immunoassay, involving two specific antibodies for the target molecules. In the first step, the hormones present in the sample bind simultaneously to: a polyclonal anti-hormone antibody, labeled with acridinium ester and a monoclonal anti-hormone antibody, immobilized on paramagnetic particles. After incubation, the formed complexes were captured on the solid phase, and excess unbound reagents were removed by washing. The chemiluminescent reaction was initiated by adding an acidic reagent followed by a basic reagent, leading to the oxidation of acridinium ester, which generates a light emission directly proportional to the hormone concentration in the sample.

Quantification limits of the assay: FSH: 0.3 mIU/mL; estradiol: 11.8 pg/mL; LH: 0.07 mIU/mL; progesterone: 0.23 ng/mL

Results

For compounds without available analytical standards, presumptive identification was performed by high-resolution MS/MS analysis, based on comparison of fragmentation patterns with spectral databases. The monitored ions and the fragments used for confirmation are listed in Table S1.

Table S1. Chemical compounds qualitatively identified by UHPLC-HRMS/MS

Compound	Chemical formula	Monitored ion $[M-H]^-$	Retention time (min)	Fragments
Eupafolin	$C_{16}H_{12}O_7$	315.0510	15.64	136.9884; 228.0414; 300.0269
Acid hidroxiferulic	$C_{16}H_{20}O_{10}$	371.0983	8.73	121.0638; 165.0544; 166.0576
Acid malic	$C_4H_6O_5$	133.0134	0.7	115.0028; 89.0249
Acid azelaic	$C_9H_{16}O_4$	187.0976	13.99	97.2032; 125.1052
Acid carnosic	$C_{20}H_{28}O_4$	331.1915	23.44	287.2015
Carnosol	$C_{20}H_{26}O_4$	329.1758	21.00	285.1856; 201.0897
Metil-carnosat	$C_{21}H_{30}O_4$	345.2071	24.00	96.95904; 286.1950; 309.17545
Rosmadial	$C_{20}H_{24}O_5$	343.1551	20.37	343.1564; 299.16647
Rosmanol	$C_{20}H_{26}O_5$	345.1707	17.15	268.1477; 283.1701; 301.1800
Isorosmanol/epirosmanol	$C_{20}H_{26}O_5$	345.1707	16.65/19.02	301.1781; 231.1014; 203.1041
7-Metoxi-rosmanol	$C_{21}H_{28}O_5$	359.1864	20.29	227.1074; 283.1704; 329.1757
Ursolic acid	$C_{30}H_{48}O_3$	455.3530	25.26	293.18048; 455.3530; 456.35843
Cirsimarin	$C_{23}H_{24}O_{11}$	475.1246	15.36	197.0584; 283.0239; 313.0708
Acid salvianolic B	$C_{36}H_{30}O_{16}$	717.1461	13.38	475.1007; 295.0594; 277.0490
Acid salvianolic C	$C_{26}H_{20}O_{10}$	491.0983	15.18	311.05740; 293.1258
Salvinorin A	$C_{23}H_{28}O_8$	431.1711	18.43	373.1617; 341.1357; 313.1392; 267.1339
Salvinorin B	$C_{21}H_{26}O_7$	389.1606	18.04	373.1599; 295.1299; 267.1339
Salvigenin	$C_{18}H_{16}O_6$	327.0874	15.68	296.0676; 268.0732; 240.0772; 133.0639
Vitexin (apigenin 8-C-glucoside)/isovitexin	$C_{21}H_{20}O_{10}$	431.0983	13.33	341.05179; 339.14789; 269.04587
Kaempferol-3-O-rutinoside	$C_{27}H_{30}O_{15}$	593.1512	9.36	299.05615; 284.03281; 227.0341
Kaempferol-O-glucoside	$C_{21}H_{20}O_{11}$	447.0933	12.14	284.04077; 284.03299; 255.02995
Pratensein	$C_{16}H_{12}O_6$	299.0561	16.78	284.03284; 283.02505; 257.04111
Irisolidone	$C_{17}H_{14}O_6$	313.0717	17.73	298.04849; 269.04581; 165.01836
Procyanidine B1/B2	$C_{30}H_{26}O_{12}$	577.1351	9.17	125.0240; 289.0698; 407.0802
Cyanidin 3-O-glucoside	$C_{21}H_{21}ClO_{11}$	483.0699	12.15	241.0508; 285.0404; 465.1033
Quecitin 3-O glucuronid	$C_{21}H_{18}O_{13}$	477.0674	10.33	151.0062; 301.0353
Quercetrin	$C_{24}H_{22}O_{15}$	549.0886	9.54	300.0042; 505.0595

Epigallocatechin gallate	$C_{22}H_{18}O_{11}$	457.0776	15.64	169.0143; 305.0668
Hispidulin	$C_{16}H_{12}O_6$	299.0561	16.78	136.9876; 284.0331
Hispidulin glucuronide	$C_{22}H_{20}O_{12}$	475.0882	13.35	299.056; 284.0327

Identification of the specific compounds in sage extract was done using HRMS Figures S5 and S6

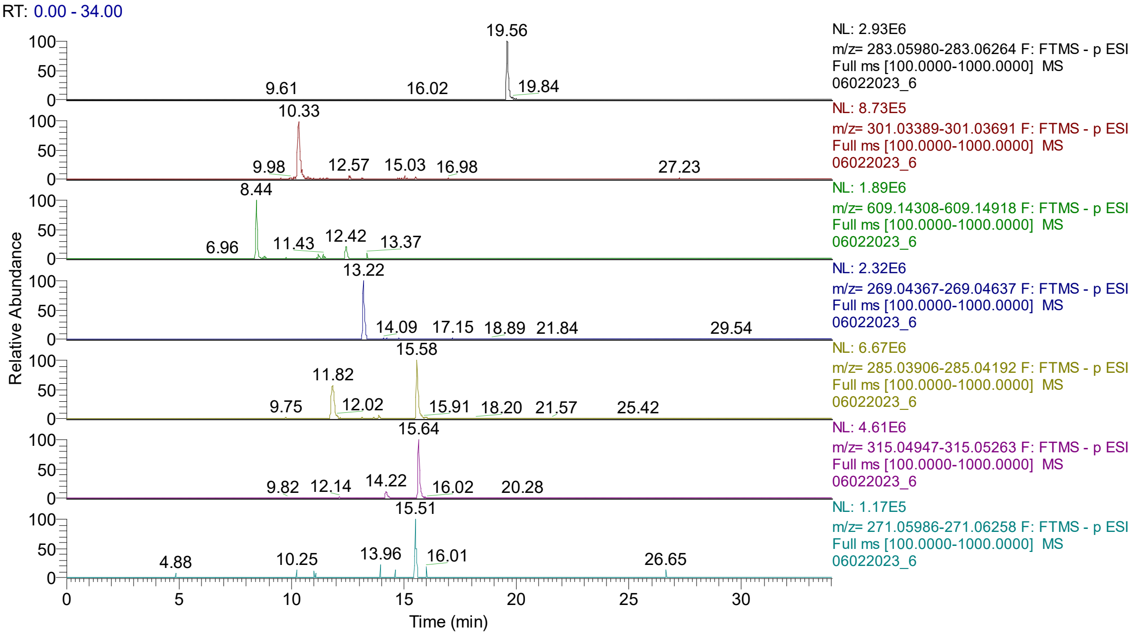

Figure S5. LC-HRMS chromatogram of the sage extract, in which the following compounds were identified: glycitein (m/z 283.0612, TR 19.56); quercetin (m/z 301.0354, TR 10.33); rutin (m/z 609.14613, TR 8.44); apigenin (m/z 269.04502, TR 13.22); luteolin and kaempferol (m/z 285.04049, TR 11.82 and 15.58, respectively); methoxy-luteolin (m/z 315.05105, TR 15.64); naringenin (m/z 271.0612, TR 15.51). Chromatograms were extracted from TIC using a 5 ppm window.

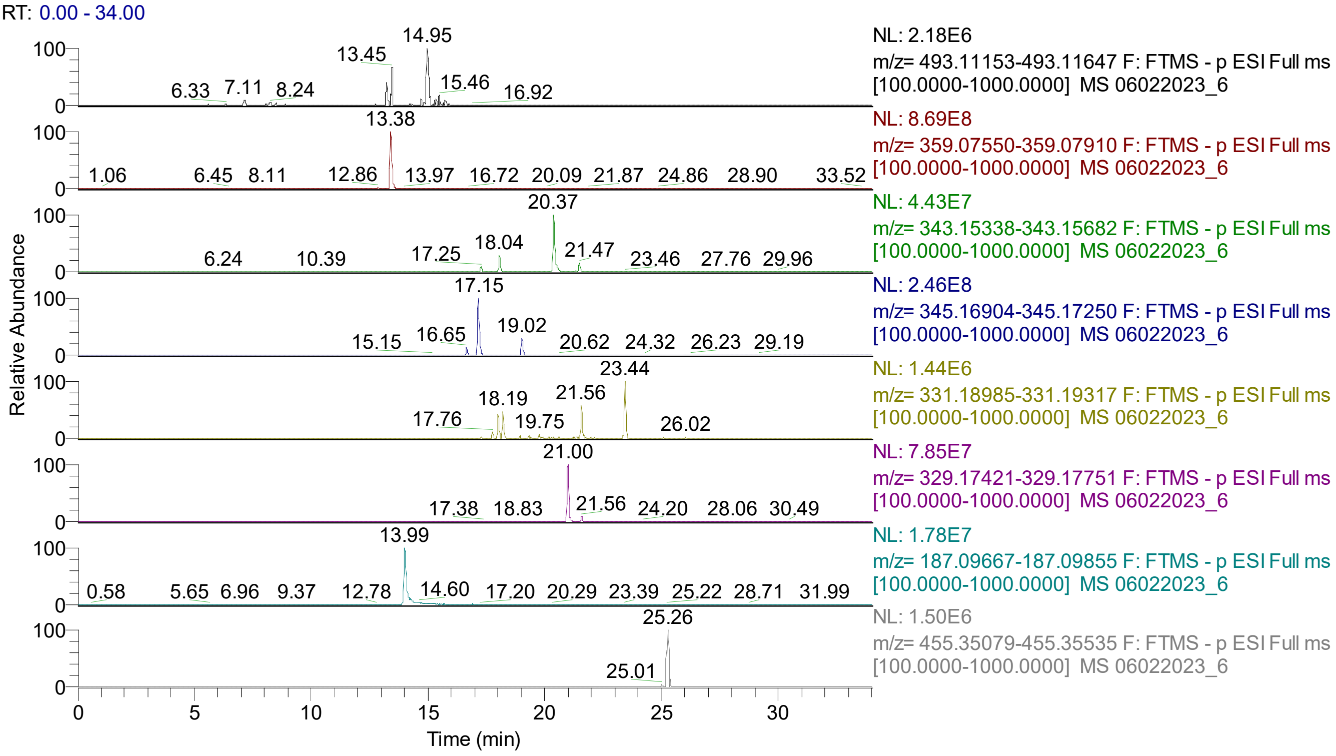

Figure S 6 . The LC-HRMS chromatogram for the sage extract identified the following compounds: salvianolic acid A (m/z 493.11401, TR 14.95), rosmarinic acid (m/z 359.0772, TR 13.38), rosmanol (m/z 345.17077, TR 17.15), carnosic acid (m/z 331.1915, TR 23.44), carnosol (m/z 329.17585, TR 21.00), azelaic acid (m/z 187.0976, TR 13.99), and ursolic acid (m/z 455.3530, TR 25.26). The chromatograms were extracted from TIC (Total Ion Chromatogram) using a 5 ppm window.

Confirmation was done by identify the fragmentation patern in MS-MS analysis (Figure 7S)

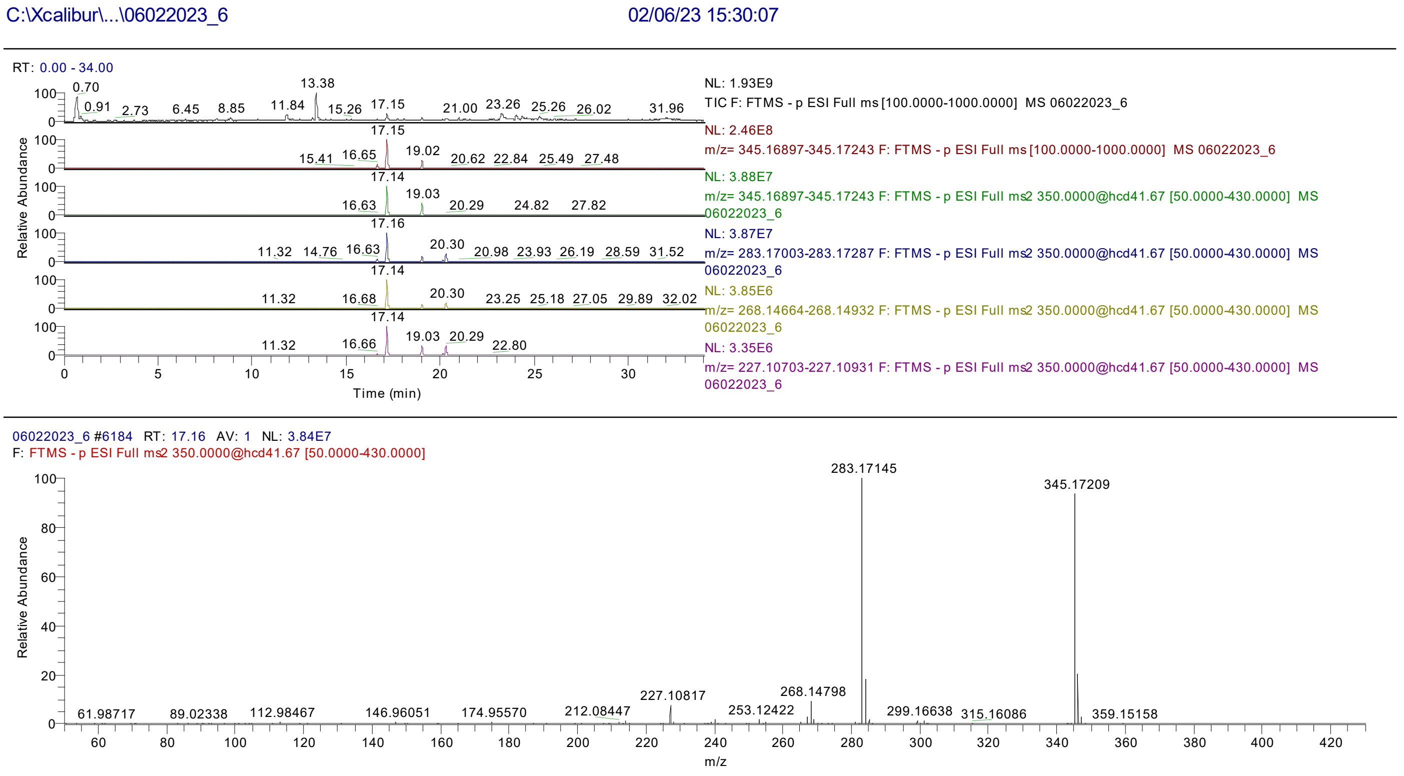

Figure S7. Full MS and MS-MS chromatogram for the sage extract, where rosmanol was identified. From top to bottom: TIC (Total Ion Chromatogram) showing the identified compound at 17.15 min, full MS chromatogram for the extracted mass m/z 345.17, MS-MS chromatogram for the precursor ion with the mentioned mass, and the characteristic ionic fragments m/z 283.17, 268.14, and 227.10 (TR 7.14), identified as rosmanol, according to MassBank. The chromatograms were extracted from TIC using a 5 ppm window.

Table S2. Effect of metformin, the synthetic compound, and sage on body weight (G) of rats with induced diabetes at different treatment time points* Data are presented as median values per group and time point, due to the non-normal distribution of the variable

Weight (g)	Group 1 (L1) control	Group 2 (L2) untreated	Group 3 (L3) metformin-treated	Group 4 (L4) treated with S	Group 5 (L5) sage -treated	Group 6 (L6) treated with S and sage
week 1	230	228	230	229	198	204
week 2	240	246	240	243	226	261
week. 3	257	246	257	251	240	261
week 4	261	259	261	260	237	245
week 5	263	259	263	261	237	245
week 6	263	255	263	259	248	244
week 7	265	252	265	258	259	271
week 8	270	217	270	243	228	252

*The value is expressed as mean ± standard deviation (n = 5).

Table S3. Effect of metformin, the synthetic compound, and sage on blood glucose levels in rats with induced diabetes at different treatment time points *. Data are presented as median values per group and time point, due to the non-normal distribution of the variable

Glucose level mg/dL	Group 1 Control (L1)	Group 2 Untreated (L2)	Group 3 metformin-treated (L3)	Group 4 treated with S (L4)	Group 5 sage-treated (L5)	Group 6 treated with S and sage (L6)
week 1	100	109	109	98	99	106
week 2	99	424	424	600	501	255
week. 3	98.9	374	388	570	578	500
week 4	97.5	382	365	500	306	326
week 5	97.85	392	350	420	459	600
week 6	99.225	456	268	410	432	600
week 7	98.5	558	470	504	574	571
week 8	114	394	112	209	340	121

*The value is expressed as mean ± standard deviation (n = 5).

Table S4. Summary of pairwise comparisons of blood glucose level between treatment groups over time using Kruskal–Wallis test followed by Dunn's post-hoc analysis with Bonferroni correction (α = 0.0033)

week	Groups Compared	p-value (Dunn)	Significant (Bonferroni corrected significance level: 0.0033)
2	L1 vs L2	0.0354	no
	L1 vs L3	0.0050	no
	L1 vs L4	0.0024	yes
	L1 vs L5	0.0084	no
3	L1 vs L4	0.0009	yes
	L1 vs L5	0.0029	yes
	L1 vs L6	0.0018	yes
	L2 vs L4	0.0423	no
4	L1 vs L3	0.0362	no
	L1 vs L4	0.0015	yes
5	L1 vs L2	0.0141	no
	L1 vs L4	0.0250	no
	L1 vs L5	0.0136	no
	L1 vs L6	0.0000	yes
	L3 vs L6	0.0024	yes
6	L1 vs L2	0.0152	no
	L1 vs L4	0.0075	no
	L1 vs L5	0.0129	no
	L1 vs L6	0.0002	yes
	L3 vs L6	0.0070	no
7	L1 vs L2	0.0128	no
	L1 vs L3	0.0474	no
	L1 vs L4	0.0073	no
	L1 vs L5	0.0062	no
	L1 vs L6	0.0087	no
8	L1 vs L2	0.0041	no
	L1 vs L5	0.0096	no
	L2 vs L3	0.0260	no
	L2 vs L6	0.0164	no
	L5 vs L6	0.0401	no
